# Mouse visual cortex areas represent perceptual and semantic features of learned visual categories

**DOI:** 10.1038/s41593-021-00914-5

**Published:** 2021-09-20

**Authors:** Pieter M. Goltstein, Sandra Reinert, Tobias Bonhoeffer, Mark Hübener

**Affiliations:** 1 Max Planck Institute of Neurobiology, Martinsried, Germany; 2 Graduate School of Systemic Neurosciences, Ludwig-Maximilians-Universität München, Martinsried, Germany

**Keywords:** Visual system, Learning and memory, Cortex, Sensory processing

## Abstract

Associative memories are stored in distributed networks extending across multiple brain regions. However, it is unclear to what extent sensory cortical areas are part of these networks. Using a paradigm for visual category learning in mice, we investigated whether perceptual and semantic features of learned category associations are already represented at the first stages of visual information processing in the neocortex. Mice learned categorizing visual stimuli, discriminating between categories and generalizing within categories. Inactivation experiments showed that categorization performance was contingent on neuronal activity in the visual cortex. Long-term calcium imaging in nine areas of the visual cortex identified changes in feature tuning and category tuning that occurred during this learning process, most prominently in the postrhinal area (POR). These results provide evidence for the view that associative memories form a brain-wide distributed network, with learning in early stages shaping perceptual representations and supporting semantic content downstream.

## Abstract (web summary)

Goltstein et al. investigate the role of mouse visual cortical areas in information-integration category learning. They report widespread changes in neuronal response properties, most prominently in a higher visual area, the postrhinal cortex.

## Main

Categorization involves associating multiple stimuli based on perceptual features, functional (semantic) relations or a combination of both^1,2^. Learned category representations help animals and humans to react to novel experiences because they facilitate extrapolation from knowledge already acquired^3,4^. Learning and recalling of categories activates a large number of brain areas, including sensory cortical regions, highlighting the associative nature of these representations^5,6^. However, it is unknown whether the formation of a neuronal category representation occurs in all of these activated brain areas jointly, or whether it is stored only in a subset of higher cortical association areas.

In primates, single-neuron correlates of category selectivity have been found in many cortical regions. In areas such as prefrontal cortex, lateral intraparietal cortex, posterior inferotemporal cortex and the frontal eye fields, substantial populations of category-selective neurons were observed following category learning^7–9^. Neural correlates are present at intermediate processing stages, for instance in inferotemporal cortex, but were found to be more perceptually biased compared to correlates in prefrontal cortex^10,11^. In contrast, primate sensory areas (for example, middle temporal area (MT)﻿ and V4) altogether show little category selectivity^12,13^. This brain-wide pattern appears similar to that of choice probability, the covariation of a neuron’s activity fluctuation with behavioral choice^14,15^, which, as a recent model suggested, can drive plasticity resulting in neurons becoming more category-selective^16^. This model might explain why there are few, if any, observations of category selectivity in lower visual areas, despite neurons’ often exquisite tuning for the visual stimuli to be categorized, such as oriented gratings^17,18^.

Nevertheless, certain studies indicate that sensory areas do play some role in category learning. Selectivity for low-dimensional auditory categories (for example, tone frequency) has been reported in auditory cortex^19,20^. Functional magnetic resonance imaging (fMRI) studies in humans point to a role of early visual areas V1–V3 in learning to discriminate dot-pattern categories^21^ and iso-oriented bars^22^, suggesting that these areas might be involved in perceptual disambiguation of stimuli belonging to different categories. A recent behavioral study in humans reports a role for early, retinotopically organized, visual areas in a perceptually challenging category learning task that requires simultaneous weighing of multiple feature dimensions, that is, information integration^23^. While these findings may seem at odds with results from single-unit recordings in monkeys, it is possible that the contribution of visual cortex to category learning depends on rather subtle changes in a restricted set of neurons. Such changes might serve to enhance feature selectivity supporting perceptual discrimination of the stimuli to be categorized and would go undetected without knowing neurons’ tuning curves before learning.

Here we use mice to investigate how early cortical stages of visual information processing are involved in learning and representing visual categories. We show that they can perform information-integration category learning and that this behavior depends, in part, on retinotopically selective visual cortex neurons. Using long-term two-photon calcium imaging, we detail response properties of large groups of neurons across nine areas of the mouse visual cortex throughout category learning. We find that learning results in newly acquired neuronal responses to choice and reward, but also in changes in stimulus and category tuning that support enhanced discrimination of learned visual categories.

## Results

### Mice discriminate, generalize and memorize visual categories

**Fig. 1:**
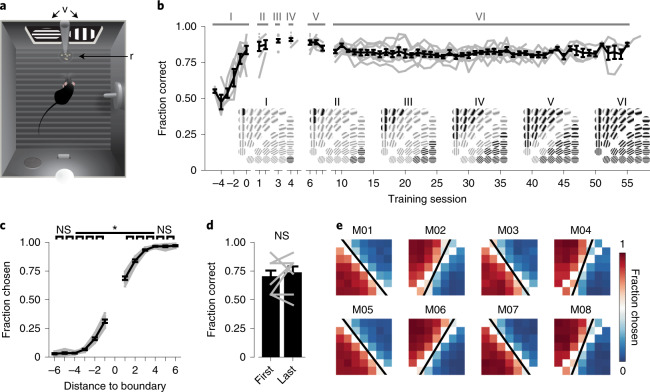
Mice learn discriminating information-integration categories in a touch screen operant chamber. **a**, Behavioral chamber, from above. Touch screens (v) display visual stimuli and record screen presses; food pellet rewards (r) are delivered via a pellet feeder into a dish. A drinking bottle, house light, speaker, and lever are positioned on the east and south walls. **b**, Mean (±s.e.m.) learning curve. Gray lines represent individual animals (*n* = 8 mice). Latin numerals denote category training stages. Insets show active stimuli (black) and not-yet-introduced stimuli (gray). **c**, Fraction of stimuli chosen as a function of the stimulus’ distance to the category boundary (black lines denote the mean ± s.e.m., and gray lines represent individual mice; two-sided Kruskal–Wallis test, H_(11) _= 90.8, *P* = 1.17 × 10^−14^, post hoc two-sided Wilcoxon matched-pairs signed-rank (WMPSR) test; *n* = 8 mice). **d**, Fraction of correct trials for novel and familiar stimuli. ‘First’ represents the mean (±s.e.m.) performance on the first trial of newly introduced stimuli at stages V and VI (only for first training session of each stage). ‘Last’ represents the performance for the same stimuli but in the first trials of the last training sessions of stages V and VI. Gray lines represent individual mice (two mice had identical performances at 0.8; two-sided WMPSR test: W = 5, *P* = 0.50; *n* = 8 mice). **e**, The fraction of trials on which a stimulus was chosen (data from stage VI) for each mouse (M01–M08). White tiles diagonally intersecting plots stand for stimuli directly on the category boundary, which were not shown. The black line represents the fitted, behaviorally expressed category boundary. NS (not significant), *P* > 0.05; *P < 0.05.

**Extended Data Fig. 1:**
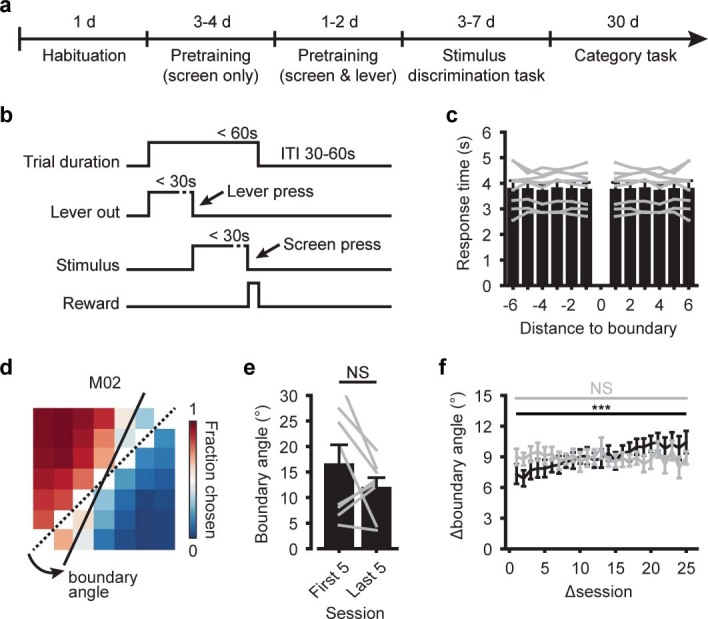
Category learning in a touch screen operant chamber. **a**, Training stages of category learning using touch screen operant chambers (Methods). **b**, Sequence of events in a single trial. ITI: Intertrial interval. **c**, Time between lever press and screen press, as a function of the stimulus’ distance to the category boundary. Bars show mean (±s.e.m.; *n*=8 mice), gray lines show data of individual animals. **d**, Example showing category boundary angles. The dashed line indicates the trained category boundary and the solid line indicates the individually learned category boundary. The boundary angle is defined as the absolute minimum angle between the two lines. **e**, Mean (±s.e.m.) boundary angle of all mice, for the first five (daily) training sessions of stage ‘VI’ and the last five sessions of stage ‘VI’ (two-sided WMPSR test, W=9, *P*=0.25; *n*=8 mice). Gray lines show individual mice. **f**, Between-session change in boundary angle (Δboundary angle; mean ±s.e.m.) as a function of how closely sessions were spaced in time (Δsession; two-sided Kruskal-Wallis test, H_(24)_=57.1, *P*=1.6·10^−4^; *n*=8 mice). Gray line shows the same data, but with shuffled session order (two-sided Kruskal-Wallis test, H_(24)_=31.6, *P*=0.14; *n*=8 mice). All panels: NS (not significant) *P*>0.05, *** *P*<0.001).

To test the ability of mice to learn visual categories, we trained eight male mice in a touch screen operant chamber to discriminate a set of 42 grating stimuli that differed in orientation and spatial frequency (Fig. 1a and Extended Data Fig. 1a,b). The two-dimensional (2D) stimulus space was divided by a diagonal category boundary into a rewarded and a non-rewarded category (Fig. 1b). Such information-integration categories^24^ are characterized by the requirement to weigh multiple stimulus feature dimensions, here spatial frequency and orientation, simultaneously. By design, the categorization task has a perceptual component (discrimination of orientation and spatial frequency) and a semantic component (multiple stimuli sharing the same meaning). Learning this task is akin to, for example, learning to distinguish paintings from Rembrandt and Vermeer, two 17th century Dutch painters whose paintings differ in subtle features like aspects of the underlying geometry and lighting (see also ref. ^25^). First, animals were trained over a period of 4 to 6 d to discriminate two stimuli that were maximally distant from the category boundary (Fig. 1b; stage I). Once mice discriminated these initial stimuli well above chance, additional stimuli were introduced, progressively closer to the category boundary until the animals reached stage VI, in which all stimuli belonging to both categories were presented. The performance of all mice stayed above chance throughout, even though 40 new stimuli were added over a period of only 6 to 8 d.

Categorization behavior has two main components: sharp discrimination of stimuli across a category boundary and generalization of stimuli within a category. The choice behavior of trained mice reflected both of these components: stimuli that were closer to the category boundary were well discriminated (that is, stimuli introduced at stages III to VI; Fig. 1c and Extended Data Fig. 1c), while stimuli that were more distant from the category boundary were all chosen (or rejected) with a similar probability (that is, stimuli introduced at stages I to III). Mice also readily extrapolated their behavior to novel stimuli: the average performance on the first trials showing stimuli of stages V and VI did not significantly differ from the performance on similar first trials showing the same stimuli in the final training sessions of these stages (we tested only stages V and VI as the comparison required multiple sessions per stage; Fig. 1d). Altogether, our results demonstrate that mice differentiate stimuli across the category boundary, generalize stimuli within categories and extend this behavior to stimuli that had not yet been encountered before.

While all mice were trained to discriminate the 2D stimulus space using a category boundary with an angle of 45°, the learned boundary angle of individual animals often deviated somewhat from the trained boundary (Fig. 1e and Extended Data Fig. 1d). This phenomenon is known as attentional bias or rule bias^26,27^ (but see also ref. ^28^ and Methods) and indicates that animals had a tendency to categorize according to one stimulus dimension, here grating orientation. To our surprise, the observed deviation in angle from the trained category boundary did not significantly decrease with further training (Extended Data Fig. 1e). Instead, the boundary angle gradually and slightly shifted, as reflected by a significantly higher similarity between consecutive days compared to periods spaced more than 20 d apart (Extended Data Fig. 1f). This implies that the mismatch between the trained and the individually learned category boundary reflected, to some extent, a mnemonic aspect, and not only day-to-day inaccuracies.

Thus, mice learned to discriminate a large set of visual stimuli by generalizing existing knowledge using an individually learned categorization strategy, which was remembered across many days. In other words, mice had formed a semantic memory.

### Learned visual categorization partially depends on plasticity in visual areas

**Fig. 2:**
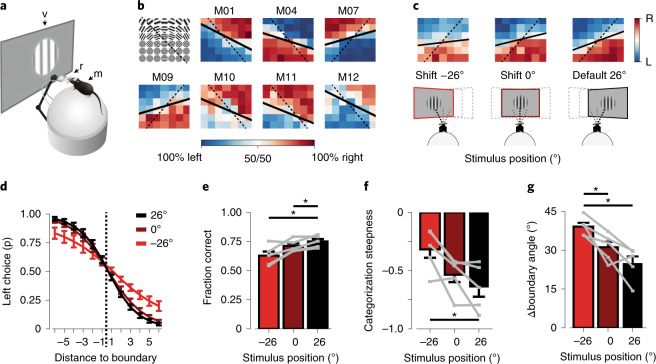
Head-fixed category learning depends on plasticity in early visual areas. **a**, Head-fixed conditioning setup. A head-fixed mouse (m) is placed on an air-suspended Styrofoam ball, facing a computer monitor (v). Two lick spouts (r) in front of the mouse supply water rewards and record licks. **b**, The fraction of left/right choices of seven mice (data of stages II to VI). The grid (top left) shows an example stimulus space. Solid lines indicate fitted, individually learned category boundaries, while dashed lines indicate trained boundaries. **c**, Illustrations (bottom) showing systematic displacement of the stimulus position by repositioning the monitor. Per-stimulus category performance (top), as in **b**, but for different stimulus positions (averaged across training sessions and mice). The default stimulus position used during preceding category training was at 26° azimuth. For display purposes, the grids are flipped such that the stimulus-to-category mapping is similar across animals (top left, ‘lick-left’; bottom right, ‘lick-right’). **d**, Sigmoid fit of the fraction of left choices (mean ± s.e.m. across mice; *n* = 5), as a function of the stimulus’ distance to the category boundary for different stimulus positions. **e**, Fraction of correct trials for default (26°) and shifted (0°, −26°) stimulus positions (mean ± s.e.m. across mice, *n* = 5; gray lines represent individual mice; two-sided Kruskal–Wallis test, H_(2) _= 6.1, *P* = 0.046; post hoc one-sided WMPSR test, −26° versus 26°: W = 0, *P* = 0.031; 0° versus 26°: W = 0, *P* = 0.031; *n* = 5 mice). **f**, As in **e**, for categorization steepness (of the sigmoid fit; two-sided Kruskal–Wallis test, H_(2) _= 6.0, *P* = 0.049; post hoc one-sided WMPSR test, −26° versus 26°: W = 15, *P* = 0.031; *n* = 5 mice). **g**, As in **e**, for the boundary angle difference between trained and individually learned category boundaries (two-sided Kruskal–Wallis test, H_(2) _= 9.1, *P* = 0.011; post hoc one-sided WMPSR test, −26° versus 26°: W = 15, *P* = 0.031; −26° versus 0°: W = 15, *P* = 0.031; *n* = 5 mice). *P < 0.05.

**Extended Data Fig. 2:**
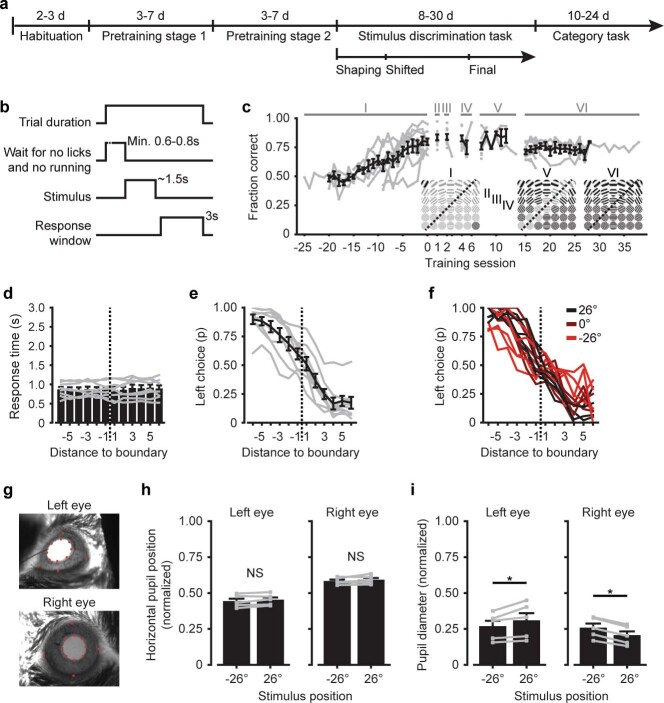
Category learning in a head-fixed operant conditioning setup. **a**, Timeline showing training stages for head-fixed category learning (Methods). **b**, Trial sequence. **c**, Mean (±s.e.m.; *n*=8 mice) learning curve for head-fixed category learning. Gray represents individual animals. Category training stages are marked by Latin numerals. Insets show example stimulus spaces, with stimuli that were included at each stage in full contrast and not-yet-introduced stimuli in gray (stages II-IV are not shown). **d**, Mean (±s.e.m.; *n*=8 mice) response time of the first lick after stimulus onset, as a function of the stimulus’ distance from the category boundary. **e**, Fraction of left choices (mean ±s.e.m.; *n*=8 mice), as a function of the stimulus’ distance from the category boundary. Gray represents individual mice, averaged across all training sessions of stage VI. **f**, As in (**e**), but for mice performing the task at different stimulus positions (*n*=5). Colors indicate the position of the monitor at which mice performed the task (default position 26°). **g**, Example images from eye tracking cameras. Red dots show automated annotations made using DeepLabCut^109,110^. **h**, Horizontal normalized pupil position (Methods) during stimulus presentation, for stimulus positions −26° (monitor shifted) and 26° (default position). Gray represents individual mice, bars show mean ±s.e.m. (one-sided WMPSR test, left eye: W=2, *P*=0.094; right eye: W=2, *P*=0.094; *n*=5 mice). **i**, as (**h**), for pupil diameter (one-sided WMPSR test, left eye: W=0, *P*=0.031; right eye: W=15, *P*=0.031; *n*=5 mice). Note that, as in humans^116^, the ipsilateral pupil contraction (that is, the pupil reflex on the side where the monitor is positioned) is stronger than the contralateral contraction. All panels: NS (not significant) *P*>0.05; * *P*<0.05.

We next implemented a head-fixed version of the categorization task that provided precise control over the visual stimulus and allowed for simultaneous two-photon microscopy. Mice were trained using similar (but not identical) stimulus spaces and training stages as described above (Fig. 2a, Extended Data Fig. 2a–c and Methods). The head-fixed task differed from the freely moving, touch screen task in two aspects. First, in each trial, only a single stimulus was shown at a specific location on the monitor. Second, the mouse had to explicitly report the category of the stimulus by licking on one of two lick spouts providing a water reward. Therefore, this task required the mouse to compare the shown stimulus to a memorized category representation. Animals took longer to learn the initial stimulus discrimination compared to the touch screen task (head-fixed, 9–25 sessions; touch screen, 4–6 sessions), but mice were able to generalize to additional stimuli at nearly the same rate in both tasks (Fig. 1b and Extended Data Fig. 2c). At the final training stage (VI; complete stimulus set) all animals discriminated and generalized stimuli according to individually learned category boundaries (Fig. 2b and Extended Data Fig. 2d,e). As in the touch screen task, animals also showed different degrees of rule bias, now favoring discrimination along the spatial frequency axis of the stimulus space (Methods).

As a first step in localizing the neuronal substrate of the learned category association, we exploited the fact that neurons in several areas of the visual cortex have well-defined, small receptive fields^18,29^. After mice had learned categorizing stimuli at a specific position in their visual field (26° azimuth), we proceeded with repeated sessions in which the stimulus position was pseudorandomly shifted horizontally in the visual field on a day-by-day basis (monitor positions 26°, 0° or −26° azimuth; Fig. 2c,d and Extended Data Fig. 2f). If visual cortex neurons were part of the learned category association, categorization performance of the mice should drop when these neurons are bypassed by presenting stimuli at locations outside their receptive fields^23,30,31^. Indeed, this is what we observed: performance was slightly, but significantly poorer when the categorization task was carried out using shifted stimulus positions (Fig. 2e). Specifically, the steepness of categorization across the boundary was reduced (steepness of the sigmoid fit over the fraction of left choices; Fig. 2f) and the individually learned category boundary showed a larger angular deviation from the trained boundary when the stimulus position was shifted (Fig. 2g). As a control, eye position was tracked continuously, and the horizontal eye position did not show a systematic adjustment to shifted stimulus positions (Extended Data Fig. 2g–i).

In summary, while the learned categorization behavior was not strictly limited to the exact visual field position of the stimulus, it was impaired by shifting the stimulus position. This suggests that visual areas store at least some amount of perceptual or semantic information about the learned categories.

### Repeated, multi-area calcium imaging throughout learning

**Fig. 3:**
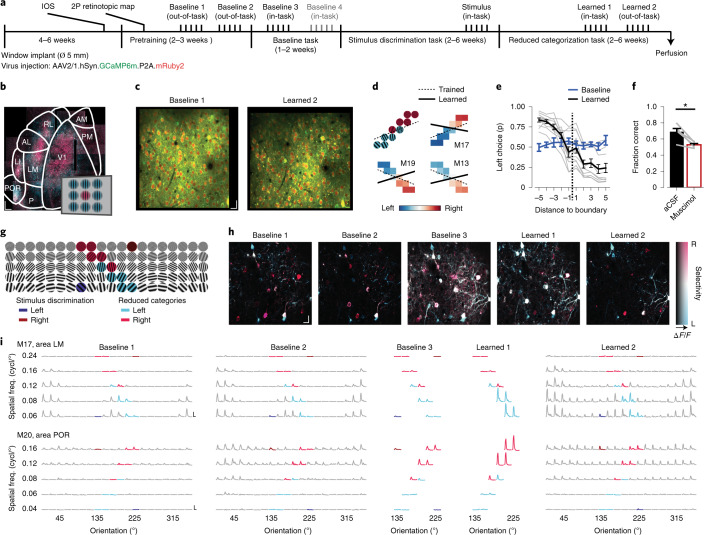
Chronic calcium imaging of multiple visual areas throughout category learning. **a**, Timeline of imaging time points (above) and stages of the behavioral paradigm (below). Each imaging time point consisted of 3–5 daily imaging sessions, in which a different area was imaged. 2P, two-photon imaging. **b**, Hue–lightness–saturation (HLS) maps showing visual cortex areas imaged using low-magnification two-photon microscopy (mouse M19). Hue represents the position of the preferred stimulus; pink denotes the center and trained stimulus location, and blue denotes eccentric stimulus locations (inset). Lightness represents response amplitude, and saturation represents selectivity. The overlaid area map is based on ref. ^37^. See Extended Data Fig. 3 for three more examples. Scale bar, 250 µm. **c**, Example FOV regions (area PM, mouse M16), acquired 110 d apart. Green, GCaMP6m; red, mRuby2. Scale bar, 20 µm. **d**, Top left, example stimuli for categorization (see also **g**). Top right and bottom, left/right choice fraction of three example mice. Dashed lines denote trained category boundaries, and solid lines denote individually learned category boundaries. Data from all ten mice are depicted in Extended Data Fig. 4b. **e**, Fraction of ‘lick-left’ choices (mean ± s.e.m. across mice; *n* = 10), as a function of the stimulus’ distance to the category boundary. Blue represents baseline time points 3 and 4. Black represents time point ‘learned 1’. Gray represents individual mice at time point ‘learned 1’. **f**, Performance (mean ± s.e.m.) after visual cortex was treated with artificial cerebrospinal fluid (aCSF; black, control) or muscimol (red, inactivation; one-sided WMPSR test, W = 15, *P* = 0.031; *n* = 5 mice). **g**, Example of full stimulus space. Dark red/blue, initial stimulus discrimination. Pink/light blue, reduced category space. **h**, HLS maps of five time points (area LM, mouse M16). Hue represents the preferred category (pink denotes right, blue denotes left), lightness the response amplitude, and saturation the selectivity (legend). Scale bar, 20 µm. **i**, Stimulus-aligned inferred spike activity across five imaging sessions for two example neurons. The *x* axes show time (−0.4 to +3.0 s around stimulus onset), and the *y* axes show response amplitude (vertical scale bars show one inferred spike per second, and horizontal scale bars show 2 s). Plots are organized into grids matching the stimulus and color space in **g**. *P < 0.05.

**Extended Data Fig. 3:**
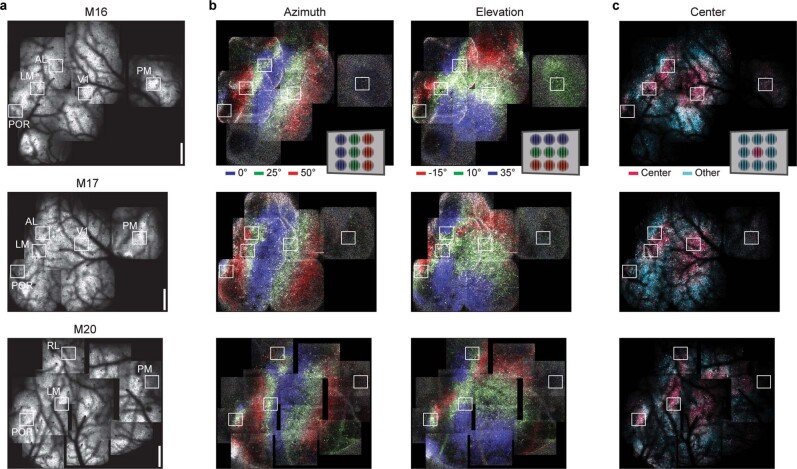
Imaging locations for three example mice. **a**, Tiled, low-magnification two-photon microscopy images showing the cortical blood vessel pattern overlaying mRuby2 fluorescence (three single-mouse examples were chosen from the dataset of 10 mice). White squares (labeled with area names) demarcate the locations of imaging regions that were followed throughout the experiment. Scale, 500 μm. **b**, Corresponding, tiled low-magnification images as in (**a**), but showing the azimuth and elevation map of primary and higher visual areas. Hue indicates the preferred stimulus (0°, 25° and 50° azimuth), lightness reflects the ΔF/F response amplitude and saturation indicates selectivity. **c**, Color-coded response maps, as in (**b**), showing the neuronal response to stimuli presented in the center of the monitor (25° azimuth, 10° elevation; approximately the position of the stimulus in the behavioral task) versus stimuli presented at surrounding positions on the monitor. Legends in the top row show the color code for preferred stimulus.

To assess in detail how the neural responses in these areas changed with category learning, we used chronic in vivo two-photon calcium imaging (GCaMP6m; Methods) to repeatedly record from the same neurons over months (Fig. 3a). We selected field-of-view (FOV) regions in cortical layer 2/3 of three to five visual areas per mouse (that is, a subselection of areas V1 (primary visual cortex), LM (lateromedial), AL (anterolateral), RL (rostrolateral), AM (anteromedial), PM (posteromedial), LI (laterointermediate), P (posterior) and POR (postrhinal); Fig. 3b), identified using intrinsic optical signal (IOS) imaging and low-magnification two-photon calcium imaging (Fig. 3b,c and Extended Data Fig. 3). This approach ensured that the imaged neurons responded to the retinotopic location of the stimulus in the behavioral paradigm.

**Extended Data Fig. 4:**
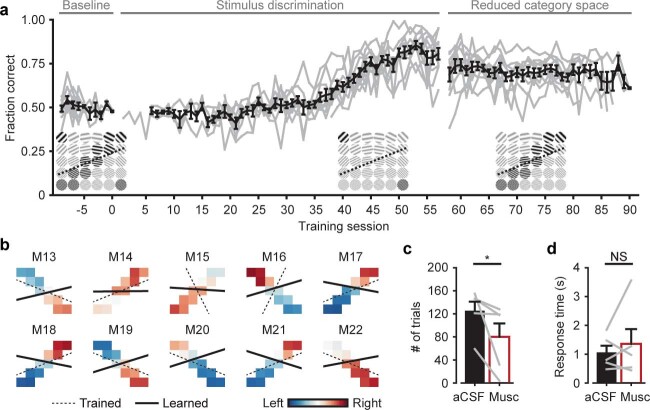
Categorization of the reduced-size stimulus space in the chronic imaging experiment. **a**, Learning curve (black, mean ±s.e.m., gray, individual mice; *n*=10) showing performance during baseline, initial stimulus discrimination and category learning. Stimuli are displayed in insets below the data (stimuli included in the corresponding stage of training are shown in black, non-included stimuli are shown in gray). Note that the animals took longer to learn initial stimulus discrimination compared to Fig. 1b and Extended Data Fig. 2c, which is possibly a consequence of the extended pre-exposure to visual stimuli during pretraining and in baseline imaging time points. **b**, Stimulus categorization of the 10 mice in the chronic imaging experiment. Color indicates the fraction left/right choices per stimulus (red, right; blue, left). Dashed lines, trained category boundary. Solid lines, individually learned category boundary. Note that the fitted boundaries might be less accurately fitted as compared to the stimulus spaces in Figs. 1 and 2, likely because of the lower number of stimuli adjacent to the boundary. **c**, Mean (±s.e.m.) number of performed (non-missed) trials under aCSF (control) and muscimol (inactivation) conditions (one-sided WMPSR test, W=15, *P*=0.031; *n*=5 mice). **d**, As (**c**), for the latency to the first lick after stimulus onset (one-sided WMPSR test, W=6, *P*=0.69; *n*=5 mice). All panels: NS (not significant) *P*>0.05, * *P*<0.05.

Within the period of chronic imaging, animals were trained to perform stimulus discrimination and, subsequently, categorization of a reduced stimulus space (Fig. 3d). These stimuli were selected from a full set of 100 possible stimuli (ten grating orientations, five spatial frequencies and two directions; Fig. 3g and Methods). As described above, categorization behavior often showed a rule bias. Therefore, we chose to train these mice on a category boundary that better aligned with this individual bias (Fig. 3d). In baseline imaging sessions, before training on the initial stimuli commenced, mice did not yet show categorization behavior. After learning, all mice categorized the stimuli in a ‘lick-left’ and a ‘lick-right’ category (Fig. 3e and Extended Data Fig. 4a), and again showed individual biases, favoring one stimulus feature over the other (Extended Data Fig. 4b).

In total, we tracked 13,019 neurons across nine visual cortical areas throughout the entire learning paradigm (Supplementary Table 1). We focused our analyses on two baseline, out-of-task time points in which tuning curves were assessed, one (or if present, two) baseline, in-task time point(s) in which behaviorally relevant visual stimuli were presented and (at chance level) discriminated, one in-task time point after category learning and a final post-learning out-of-task time point. We refer to a time series of imaging sessions of a single area in a single mouse as a chronic recording.

### Visual categorization is contingent on activity in the visual cortex

**Extended Data Fig. 5:**
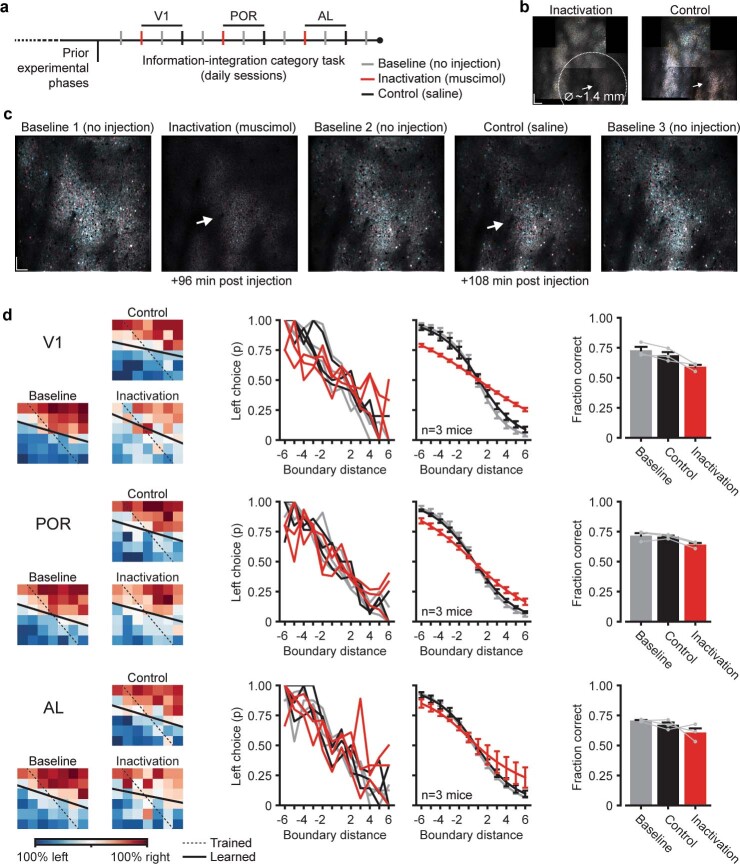
Inactivation of visual cortical areas. **a**, Timeline showing the sequence of training sessions alternating between baseline (no injection), inactivation (muscimol injection) and control (saline injection) experiments. Injections were targeted at V1, POR and AL (inactivation of AL likely also affected RL and possibly LM, inactivation of POR likely also affected LI and LM; Methods). **b**, Tiled HLS map for preferred orientation. Left, maximum spatial extent of the inhibitory effect of a muscimol injection into V1, roughly 1.4 mm in diameter. White arrow, injection location. Right, control, saline injection. Data were acquired ~130 and ~150 minutes after injection, respectively. Scale bar, 200 µm. **c**, HLS maps for preferred category, acquired during task performance, showing neuronal responses in inactivation, control and flanking baseline experiments. White arrow, injection location. Scale bar, 100 µm. **d**, Top row, data of experiments targeting V1. Left, per condition, the mean per-stimulus category choice (blue, left; red, right). Black lines, trained (dashed), and fitted, learned (solid), category boundaries. Individual mouse’s stimulus-to-category mappings were flipped for visualization (right top, ‘lick-right’; left bottom, ‘lick-left’). Middle, per condition, the fraction of left choices as function of the stimulus’ distance to the category boundary (left plot, individual mice; right plot, averaged sigmoidal fits). Right, mean (±s.e.m.) performance for each condition. Gray lines show individual mice (*n*=3). The data point ‘Baseline’ is the mean of the three baseline experiments flanking the inactivation and control experiment. Middle and bottom rows, experiments with injections targeted to areas POR and AL. Across the three areas, performance was lower after cortical inactivation as compared to the control condition (Δperformance, V1: 9.6%, POR: 6.7%, AL: 6.4%; s.d. across three mice and three areas: ±6.0%).

While previous studies have shown that, in mice, an intact visual cortex is indispensable for proper visual discrimination and detection^32,33^, it has been demonstrated that for certain visually guided behaviors subcortical structures alone are sufficient^34,35^. To test whether in our paradigm visual cortex was necessary for the correct assignment of visual stimuli to learned categories, we unilaterally silenced all visual cortical areas with the GABAergic receptor agonist muscimol. We found that this completely abolished the mice’s ability to discriminate stimuli (Fig. 3f). Importantly, the unilateral inactivation of visual areas with muscimol did not reliably abolish other task-related behaviors; three of five mice still performed a large number of trials (Extended Data Fig. 4c,d). Furthermore, targeted inactivation of specific visual cortical areas (V1, AL and POR) showed that although each of these areas contributed to visual categorization, no individual area was critically necessary (Extended Data Fig. 5). Thus, either perceptual or semantic aspects of categorization behavior, but not generalized operant behavior and motor behavior, were contingent on neuronal activity in visual cortical areas.

**Fig. 4:**
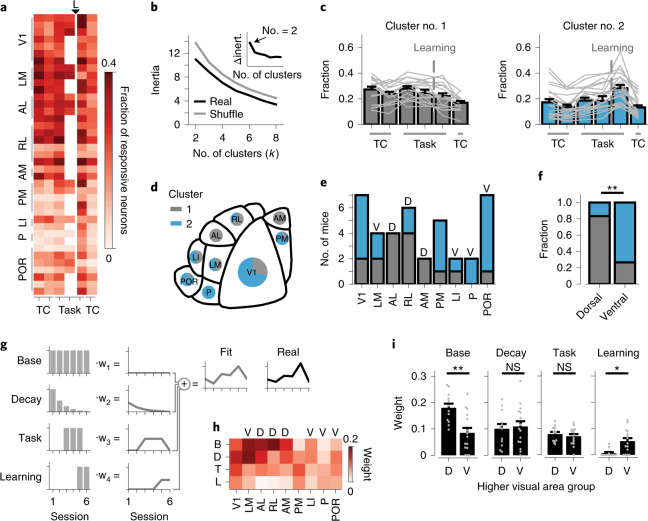
Changed fractions of stimulus- and task-activated neurons after category learning. **a**, Fraction of responsive neurons (corrected for variable trial numbers by subsampling). The *y* axis shows chronic recordings, and the *x* axis shows imaging time points. TC, out-of-task time points (tuning curves). Task, in-task time points. L, separates baseline from after category learning. The second in-task baseline was only acquired in a subset of mice. **b**, Inertia of *k*-means identified clusters of chronic recordings, as a function of the number of initialized clusters (*k*). Black, real data; gray, shuffled data. The inset shows Δinertia of real and shuffled data. The arrow indicates maximum Δinertia (at two clusters). **c**, Fraction of responsive neurons for each cluster of chronic recordings (gray represents cluster 1, and blue represents cluster 2). Bars show the mean (±s.e.m.), and light-gray lines show individual chronic recordings (*n* = 39 chronic recordings from ten mice). **d**, Map of mouse visual areas (based on ref. ^37^) showing the fraction of cluster 1 and cluster 2 chronic recordings per area. **e**, Number of chronic recordings per area, color coded for cluster identity. D, dorsal stream-associated area; V, ventral stream-associated area. Areas V1 and PM are equally associated with both streams and are therefore unlabeled (*n* = 39 chronic recordings from ten mice). **f**, The fraction of cluster 1 and cluster 2 recordings in dorsal stream-associated areas AL, RL and AM, and ventral stream-associated areas LM, LI, P and POR (chi-squared test, *χ*^2^_(1) _= 8.58, *P* = 0.0034; *n*_dorsal _= 12, *n*_ventral _= 15 chronic recordings from ten mice). **g**, Schematic of linear model predicting the fraction of responsive neurons per chronic recording (Methods). Individual components; base, overall non-changing fraction of responsive neurons; decay, exponential session-dependent reduction; task, increase during in-task time points; learning, increase after category learning. **h**, Weight (mean, across chronic recordings) of each component per visual cortical area (B, base; D, decay; T, task; L, learning). **i**, Mean (±s.e.m.) weight associated with each model component, separately for chronic recordings (gray dots) from dorsal and ventral stream-associated areas (two-sided Mann–Whitney *U* test; base, *U* = 30, *P* = 0.0073; decay, *U* = 89, *P* = 1.96; task, *U* = 79.0, *P* = 1.22; learning, *U* = 36, *P* = 0.013; *n*_dorsal _= 12, *n*_ventral _= 15 chronic recordings from ten mice; *P* values were calculated using Bonferroni correction for four comparisons). NS, *P* > 0.05, *P < 0.05, **P < 0.01.

**Fig. 5:**
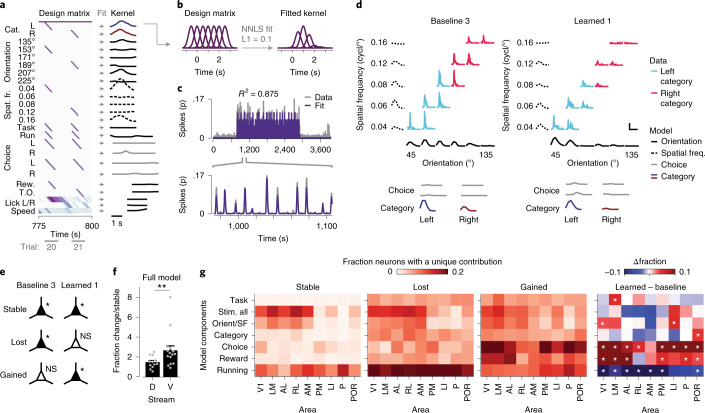
Identification of stimulus-, choice-, reward- and motor-related activity patterns of single neurons using a generalized linear model. **a**, Left, example section of a design matrix. The *y* axis shows regressor sets (cat., category; spat. fr., spatial frequency; rew., reward, T.O., time-out; Supplementary Table 3), and the *x* axis shows time and behavioral trials. A regressor set consists of multiple rows with time-shifted gaussian curves (see **b**). Right, fitted response kernels of an example neuron. **b**, Example regressor set with multiple rows of gaussian shaped curves (left). Nonnegative least squares (NNLS) fitting using L1 regularization estimated the weight of each curve (right). **c**, Inferred spike activity (gray represents data, and purple denotes the model prediction) of a single neuron for an entire imaging session. The below inset shows a zoom-in view of the model-fit that captures the amplitude and timing of inferred spiking activity. **d**, Stimulus-triggered inferred spike response of an example neuron (area POR, mouse M16) to all ten category stimuli organized in a grid of spatial frequency (*y* axis) by orientation (*x* axis). Solid lines in light blue represent the left category, and pink lines represent the right category. Vertical scale bar, 0.1 inferred spikes; horizontal scale bar, 2 s. The dashed black lines represent spatial frequency kernels, while solid black lines denote orientation kernels. The solid gray lines represent choice kernels. Dark blue/red solid lines are category kernels. Left, time point ‘baseline 3’; right, ‘learned 1’. **e**, Classification of neurons based on the significance of their modulation in the in-task time points ‘baseline 3’ and ‘learned 1’. **f**, Mean (±s.e.m.) fraction of changed (‘gained’ and ‘lost’) neurons divided by the fraction of ‘stable’ neurons in dorsal and ventral stream-associated areas. Gray dots denote individual chronic recordings (two-sided Mann–Whitney *U* test, *U* = 41, *P* = 0.009; *n*_dorsal _= 12, *n*_ventral _= 15 chronic recordings from ten mice). **g**, The fraction of neurons that showed significant unique modulation (Δ*R*^2^) by the group of regressor sets (*y* axis), separately per imaging area (*x* axis), for the groups defined in **e**. Right, difference in the fraction of neurons that showed significant unique modulation in time points ‘baseline 3’ and ‘learned 1’. White asterisks denote a significant difference from zero (chi-squared test, *P* values were calculated using Bonferroni correction for 63 comparisons). NS, *P* > 0.05, *P < 0.05, **P < 0.01.

**Fig. 6:**
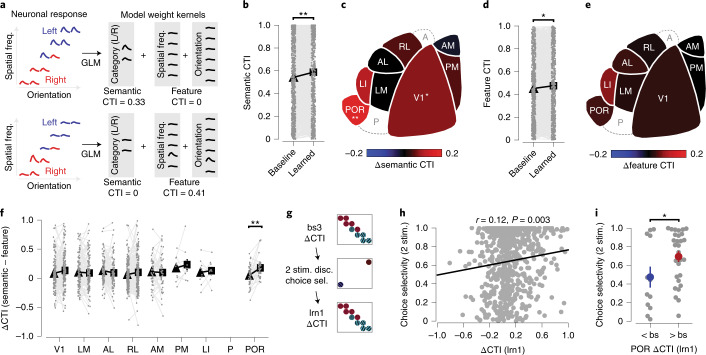
Category selectivity emerges in choice-selective POR neurons. **a**, Schematic with two examples, demonstrating that feature CTI is calculated from orientation/spatial frequency-specific weight kernels, and semantic CTI from category-specific weight kernels (obtained using the full GLM; Extended Data Fig. 8). Colored curves represent the tuning curve of a neuron (red, right-category stimuli; blue, left-category stimuli; in the grid, the *x* axis shows the orientation, and the *y* axis shows spatial frequency). **b**, Mean semantic CTI for all neurons, pooled across mice and areas, before and after category learning (two-sided WMPSR test, W = 86171, *P* = 1.83 × 10^−4^; *n* = 645 neurons from ten mice). Gray dots indicate individual neurons (Extended Data Fig. 9a). **c**, The difference in semantic CTI before (‘baseline 3’) and after (‘learned 1’) category learning, overlaid on a map of mouse visual areas (based on ref. ^37^; two-sided WMPSR test; V1, W = 4,088, *P* = 0.036; *n* = 149 neurons from seven mice; POR, W = 204, *P* = 0.009; *n* = 43 neurons from six mice; *P* values were calculated using Bonferroni correction for eight comparisons). Area P had no ‘stable’ visually modulated neurons, and area A was not imaged. **d**,**e**, As in **b** and **c**, for feature CTI (Extended Data Fig. 9b; pooled data (**d**), two-sided WMPSR test, W = 93043, *P* = 0.019; *n* = 645 neurons from ten mice). **f**, Mean ΔCTI, the difference between semantic CTI and feature CTI, before (triangle) and after (square) category learning, per visual area. Gray dots indicate individual neurons (Extended Data Fig. 9c; area POR, two-sided WMPSR test, W = 155, *P* = 9.85 × 10^−4^; *n* = 43 neurons from six mice; *P* values were calculated using Bonferroni correction for eight comparisons). **g**, Experimental timeline showing the stimulus discrimination session in which choice selectivity was determined (using the GLM), relative to sessions in which ΔCTI was calculated (‘baseline 3’ (bs3) and ‘learned 1’ (lrn1)). **h**, Correlation between choice selectivity at the time point of stimulus discrimination and ΔCTI after category learning (at ‘learned 1’). The black line represents the linear fit. Gray dots indicate individual neurons from all areas. Pearson correlation coefficient, *r* = 0.12, two-sided *P* = 0.0034; *n* = 620 neurons from ten mice. **i**, Mean (±s.e.m.) choice selectivity in POR neurons during initial stimulus discrimination, for neurons that increased (red circle) and decreased (blue circle) ΔCTI after category learning, compared to baseline (gray indicates individual neurons; two-sided, Mann–Whitney *U* test, *U* = 115, *P* = 0.047; *n*_<bs _= 12, *n*_>bs _= 29 neurons from five mice). *P < 0.05, **P < 0.01. bs, baseline.

Throughout areas of the visual cortex, we observed neuronal activity in response to visual stimulation, revealing characteristic tuning curves for orientation and spatial frequency, which, despite variability in response amplitudes, were largely stable across time points (Fig. 3g–i). To accurately describe how neuronal responses across visual cortical areas change upon category learning, we will first address the overall number of activated neurons (Fig. 4). Second, we will describe the type of information encoded by such activated neurons (Fig. 5), and finally, we will discuss to which degree stimulus-driven neurons encode the learned categories by disentangling perceptual (orientation/spatial frequency) and semantic (category) components of their tuning (Fig. 6).

### Learning recruits neurons in V1, PM and ventral stream areas

**Extended Data Fig. 6:**
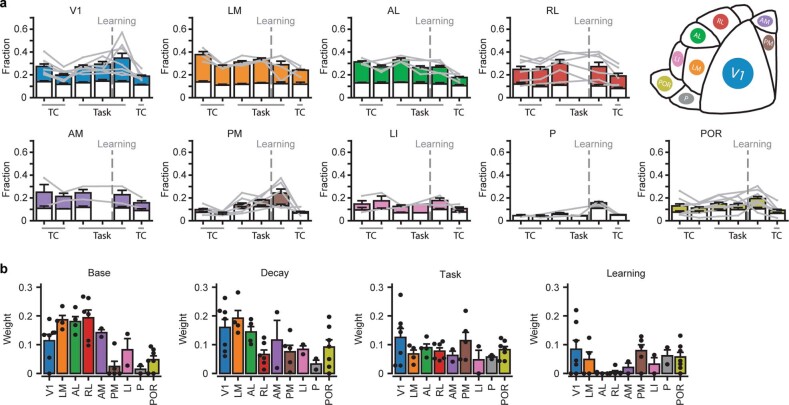
Analysis of significant stimulus- and task-responsive neurons, shown for each higher visual area individually. **a**, Bar plots depicting the mean (±s.e.m.) fraction of responsive neurons at each chronic imaging time point (*n*=39 chronic recordings from 10 mice). The white section of the bar indicates the fraction of neurons that had a significant response to only one of the stimuli, the colored section shows the fraction that responded significantly to two or more of the stimuli. Gray lines indicate data of individual chronic recordings (that is per area-mouse combination). Time points labeled ‘TC’ are out-of-task imaging sessions, time points labeled ‘Task’ are in-task imaging sessions. The vertical dashed line separates the time points before (baseline) and after category learning. Note that the second, in-task baseline time point was not imaged in a subset of experiments. Top row, right, areal color code overlaid on a schematic map of mouse higher visual areas (based on^37^). **b**, Linear model fitted weights (mean ±s.e.m.) indicating the strength by which each component contributed to the fraction of responsive neurons (*n*=39 chronic recordings from 10 mice). Colored bars show data grouped by individual areas using the color scheme in (**a**). Black dots indicate data of individual chronic recordings.

As a starting point, we explored the involvement of visual areas in category learning by comparing, across experimental time points, the fractions of neurons significantly responding during the first second of visual stimulus presentation (Methods). This approach resulted in time-varying fractions of responsive neurons for chronic recordings in all nine visual areas (Fig. 4a and Extended Data Fig. 6a). These time-varying patterns can show a signature of area-specific functional specialization for behaviorally relevant stimuli, similar to specializations for visual features, as shown in mice^36,37^ and primates^38–40^. To identify such structure without an a priori bias, we performed *k*-means clustering (Methods). First, we determined the optimal number of clusters by comparing the inertia (within-cluster sum of squares) of the clustered time-varying patterns, to the mean inertia of 100 shuffled patterns (Fig. 4b). This indicated that the time-varying patterns were best divided into two groups (Fig. 4b). To identify the difference between these two groups, we plotted their average patterns. It turned out that by far the most distinct difference was seen at the time point after learning, where cluster 2 showed a steep increase in the fraction of responsive neurons, while cluster 1 did not (Fig. 4c).

Importantly, this division of time-varying patterns of responsive neurons into two clusters largely aligned with the known areal organization of the mouse visual system. The majority of cluster 2 patterns came from areas V1, PM, P and POR, while cluster 1 patterns tended to originate from areas AL, RL and AM (Fig. 4d,e). Based on their patterns of connectivity, mouse higher visual cortical areas can be broadly subdivided into a dorsal and ventral visual stream^41,42^, akin to what has been observed in primates^43^. Grouping the areas by visual stream revealed that the cluster membership of chronic recordings systematically mapped onto the dorsal and ventral stream areal distinction (Fig. 4f).

Next, we sought to quantitatively determine which differences in the fraction of responsive neurons over time led to the separation into the dorsal and ventral stream clusters. We hypothesized that the time-varying patterns reflected multiple underlying processes with different temporal dynamics. The hypothesized components were: a stable, non-time-varying fraction of responsive neurons; an exponential decaying fraction, reflecting long-term adaptation or repetition suppression;^44,45^ an increased fraction for in-task recordings, reflecting effects of in-task attentional modulation;^46^ and a learning-related increased fraction, reflecting recruitment by learning^47^. Using linear regression, we quantified the individual contribution of each of these components, thus predicting the fraction of responsive neurons across the five (or six) time points of each chronic recording (Fig. 4g).

Investigation of the model weights revealed that the stable, non-time-varying fraction of responsive neurons was significantly larger in dorsal stream areas, indicating a larger pool of neurons that systematically responded during visual stimulus presentation (Fig. 4h,i). Importantly, there was also a clear difference in the learning-related component, which was far stronger in ventral stream areas compared to dorsal stream areas (Fig. 4h,i). Chronic recordings from V1 resembled dorsal stream areas, in that they had a large unchanged fraction of responsive neurons, but also ventral stream areas, as they were modulated by learning (Extended Data Fig. 6b). Area PM, which equally connects to dorsal and ventral stream areas^42,48^, behaved altogether similarly to ventral stream areas. We did not detect significant differences between areas or streams in the contribution of long-term adaptation and task modulation. In summary, visual category learning is associated with an increased fraction of neurons that respond during presentation of task-relevant stimuli, specifically in V1, PM and ventral stream areas.

### Learning strengthens the modulation of neurons by choice and reward

**Extended Data Fig. 7:**
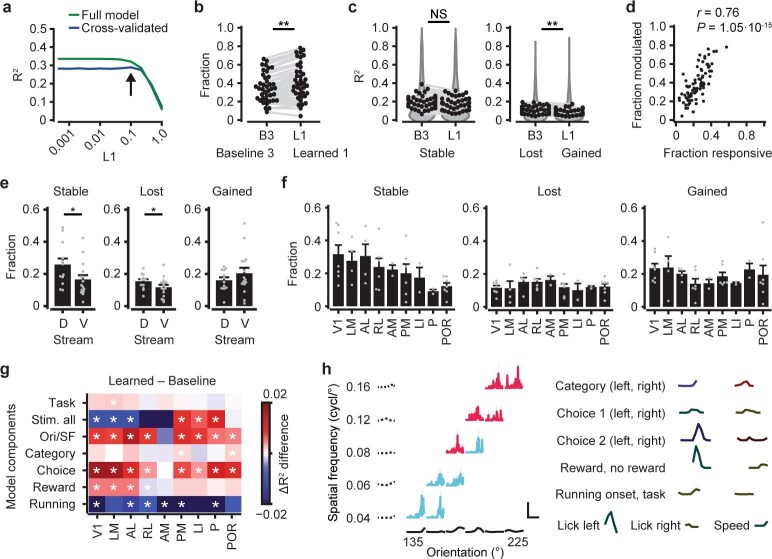
Linear model performance. **a**, Parameter tuning of the L1 value. The curves show the mean of the (larger than 0) R^2^ values across all neurons from ‘baseline 3’ and ‘learned 1’ as a function of the L1 value. Green,: full model, blue, mean of 7 cross validations. The arrow indicates a subtle bump (global maximum) just before the cross-validated R^2^ value starts to decrease, which is the optimal L1 value. **b**, Fraction of significantly modulated neurons (Methods). ‘B3’, time point ‘baseline 3’, ‘L1’, ‘learned 1’. Gray represents individual imaging regions (two-sided WMPSR test, W=195, *P*=0.0039; *n*=40 chronic recordings). **c**, Left, the R^2^ values of neurons (mean per imaging session) that were significantly modulated in both ‘baseline 3’ and ‘learned 1’ (‘stable’ neurons). Gray, individual imaging regions. Dark-gray shaded area, distribution of individual neuron R^2^ values. Right, as left, for ‘lost’ and ‘gained’ neurons (two-sided WMPSR test, ‘stable’: W=322, *P*=0.24; ‘gained’ vs ‘lost’: W=221, *P*=0.011; *n*=40 chronic recordings from 10 mice). **d**, Per imaging session, the fraction of significantly modulated neurons, obtained by the linear model, plotted against the fraction of significantly responsive neurons (based on the first second of stimulus-driven inferred spiking activity; Fig. 4 and Extended Data Fig. 6). Pearson correlation, *r*=0.7572, two-sided *P*=1.05·10^−15^; *n*=78 imaging sessions from 10 mice. **e**, The mean (±s.e.m.) fraction of ‘stable’, ‘lost’ and ‘gained’ neurons for dorsal and ventral stream associated areas (two-sided Mann-Whitney *U* test, ‘stable’: *U*=50, *P*=0.027; ‘lost’: *U*=51, *P*=0.030; ‘gained’: *U*=76, *P*=0.26; *n*_dorsal_=12, *n*_ventral_=15 chronic recordings from 10 mice). **f**, Fraction of ‘stable’, ‘lost’ and ‘gained’ neurons for each imaged area (mean ±s.e.m.; *n*=40 chronic recordings from 10 mice). Gray dots, chronic recordings from individual mice. **g**, The mean difference in ΔR^2^ before and after learning, of neurons that showed a significant unique contribution of a specific regressor group (*y* axis), per area (*x* axis). White asterisks, significant difference before and after learning (two-sided Mann-Whitney *U* test, *P*<0.05; Bonferroni corrected for 63 comparisons). **h**, Example tuning curve (area POR, mouse M16) for all 10 category stimuli of a single neuron (solid lines, blue, left category; pink, right category). Dotted black lines, spatial frequency kernels (*y* axis), solid black lines, orientation kernels (*x* axis). Right, all non-stimulus-related kernels (Methods; Supplementary Table 3). Scale bar, vertical, 0.02 inferred spikes, horizontal, 2 s. All panels: NS (not significant) *P*>0.05, * *P*<0.05, ** *P*<0.01.

What are the newly responsive neurons coding for? Recent work has shown that mouse visual cortex is functionally much more diverse than has been traditionally assumed; it can be driven and modulated by many factors beyond visual stimuli, such as running, reward and decisions^15,47,49^. We implemented a generalized linear model (GLM; Methods) to estimate the individual contributions of stimulus orientation, spatial frequency and category (that is, perceptual and semantic aspects of the visual stimulus), locomotor and licking behavior, choice and reward to the inferred spiking activity of single neurons (Fig. 5a–d and Extended Data Fig. 7a). Neurons with significant *R*^2^ values were considered modulated by the modeled factors (Methods). We limited this analysis to in-task time points (Fig. 3a) because many GLM factors were exclusive to those time points (see Supplementary Table 2 for numbers of included neurons).

Overall, we observed a slightly larger fraction of significantly modulated neurons after category learning compared to before category learning (Extended Data Fig. 7b). The *R*^2^ values of neurons that were significantly modulated both before and after learning did not change, but neurons that were significantly modulated only after learning had slightly lower *R*^2^ values compared to neurons that were only modulated before learning (Extended Data Fig. 7c). We verified that the per-session fraction of significantly GLM-modulated neurons matched the fraction of responsive neurons determined in the previous analysis (Fig. 4). Even though the latter was calculated using neuronal activity in a 1-s window after stimulus onset, while the GLM takes the entire trial into account, both values were strongly correlated (Extended Data Fig. 7d). Finally, ventral stream areas showed larger numbers of neurons that were significantly predictive only at one time point, while dorsal stream areas contained more ‘stable’ neurons (Fig. 5e,f and Extended Data Fig. 7e,f).

We quantified the unique contribution of each GLM component to explain the neuronal activity patterns (Methods). We analyzed ‘stable’ neurons that were significantly, uniquely modulated in both time points, ‘baseline 3’ and ‘learned 1’, separately from ‘lost’ and ‘gained’ neurons that were significantly, uniquely modulated either in time point ‘baseline 3’ or time point ‘learned 1’ (Fig. 5e). For each group (‘stable’, ‘lost’ and ‘gained’), we calculated the fractions of neurons that showed significant, unique modulation by each GLM component, for each area separately. Neurons in the ‘stable’ group were most prominently modulated by visual stimulus components (orientation, spatial frequency and category) and by running-related components. ‘Lost’ neurons were, in addition to being modulated by visual components, most strongly modulated by running activity. ‘Gained’ neurons, on the other hand, were, besides a modulation by visual components, modulated by behavioral choice and, to some extent, reward (Fig. 5g and Extended Data Fig. 7g). As for the category component, only area POR showed a significantly larger fraction of uniquely modulated neurons after learning. The GLM analysis therefore revealed that the gained fraction of responsive neurons could be largely attributed to an increased influence of choice and reward (see an example in Extended Data Fig. 7h), and that only in area POR more neurons contributed to the category representation. However, the model also points to a large, stable and purely stimulus-driven component within the activity pattern of many visual cortex neurons, which we investigate in the following section.

### Highly choice-selective neurons in area POR gain category selectivity

**Extended Data Fig. 8:**
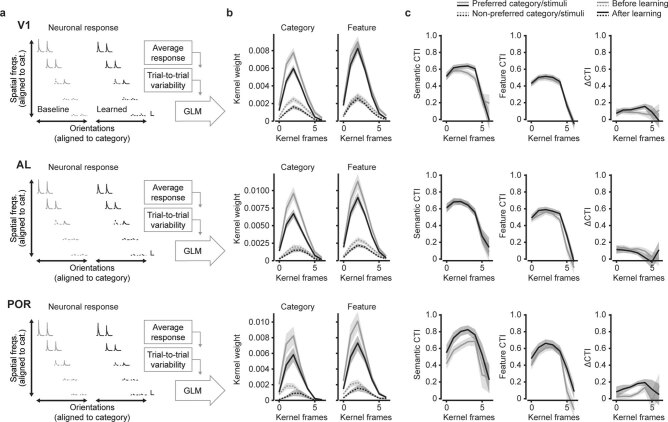
GLM weights as a function of time from stimulus onset for three example areas. Data from V1, AL and POR neurons that were significantly modulated by the visual stimulus regressors. **a**, Mean stimulus-aligned inferred spike activity of neurons that were significantly stimulus-modulated in the encoding model, shown for all 10 stimuli that were part of the learned categories. Across neurons and mice, the stimulus space was flipped such that the preferred category of each neuron was positioned left-top (gray/black solid traces), and the non-preferred category was at the right-bottom (gray/black dotted traces). The schematic on the right indicates the fitting of a GLM, resulting in weighted kernels describing how individual components of the task and the mouse’s behavior best predict the neurons’ inferred spike activity patterns. Scale bars, vertical, 0.5 inferred spikes/s, horizontal, 3 s. **b**, The mean (±s.e.m.) weight kernel associated with the preferred (solid lines) and non-preferred (dotted lines) category, averaged across neurons. The kernels were calculated using exclusively the category-specific regressors (labeled ‘Category’, left), or the orientation and spatial frequency-specific regressors (labeled ‘Feature’, right). The in-task baseline session (‘baseline 3’) is depicted in gray, the in-task session after categories were learned (‘learned 1’) is shown in black. The kernel frames were spaced 500 ms apart in time. **c**, Columns show semantic CTI, feature CTI and ΔCTI per kernel frame (mean ±s.e.m.; Methods; Fig. 6a).

Identifying category-selective neurons in visual areas is complicated by the fact that already before learning, neurons are tuned to category-defining features (such as orientation and spatial frequency). Therefore, we analyzed how model-derived tuning parameters changed with learning (including only ‘stable’ neurons, that is, significantly modulated by visual stimulus components in the full model, before and after category learning). We calculated a category tuning index (CTI) based on either category-specific model components (semantic CTI) or exclusively orientation-specific and spatial frequency-specific model components (feature CTI; using weights of the full model; Methods, Fig. 6a and Extended Data Fig. 8). The semantic CTI captures selectivity for categories that are shared across all stimuli belonging to each category and relatively independent of orientation and spatial frequency tuning. Feature CTI, on the other hand, reflects category selectivity that can be explained directly from a neuron’s tuning to orientation and spatial frequency components. As the model fits neuronal responses per trial and frame, we note that the weights used for calculating CTI reflect both the amplitude and reliability of the associated neuronal response.

**Extended Data Fig. 9:**
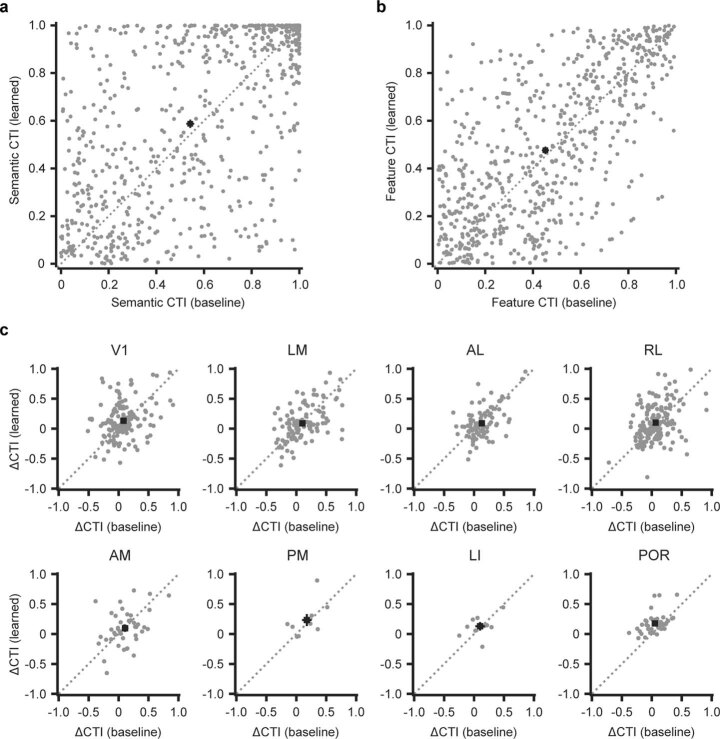
Scatter plots of semantic CTI, feature CTI and ΔCTI before and after learning. **a**, Gray dots show the semantic CTI of all neurons that were significantly stimulus-modulated before and after learning, as determined using the GLM analysis (‘stable’ neurons). Black squares and error bars show the mean (±s.e.m.; two-sided WMPSR test, W=86171, *P*=1.83·10^−4^; *n*=645 neurons from 10 mice). The *x* axis shows semantic CTI before learning (‘baseline 3’) and the *y* axis shows semantic CTI after learning (‘learned 1’). **b**, As (**a**), for feature CTI (two-sided WMPSR test, W=93043, *P*=0.019; *n*=645 neurons from 10 mice). **c**, As (**a**), each panel now shows the ΔCTI of a single visual cortical area (two-sided WMPSR test, V1: W=4914, *P*=1.61; *n*=149 neurons from 7 mice; LM: W=3305, *P*=3.86; *n*=119 neurons from 4 mice; AL: W=1809, *P*=0.46; *n*=96 neurons from 4 mice; RL: W=6545, *P*=0.68; *n*=175 neurons from 6 mice; AM: W=431, *P*=4.90; *n*=43 neurons from 2 mice; PM: W=26, *P*=7.38; *n*=10 neurons from 3 mice; LI: W=24, *P*=6.16; *n*=10 neurons from 2 mice; POR: W=155, *P*=9.85·10^−4^; *n*=43 neurons from 6 mice; *P* values are Bonferroni corrected for 8 comparisons). Note that area P is not shown because it did not contain ‘stable’ neurons.

**Extended Data Fig. 10:**
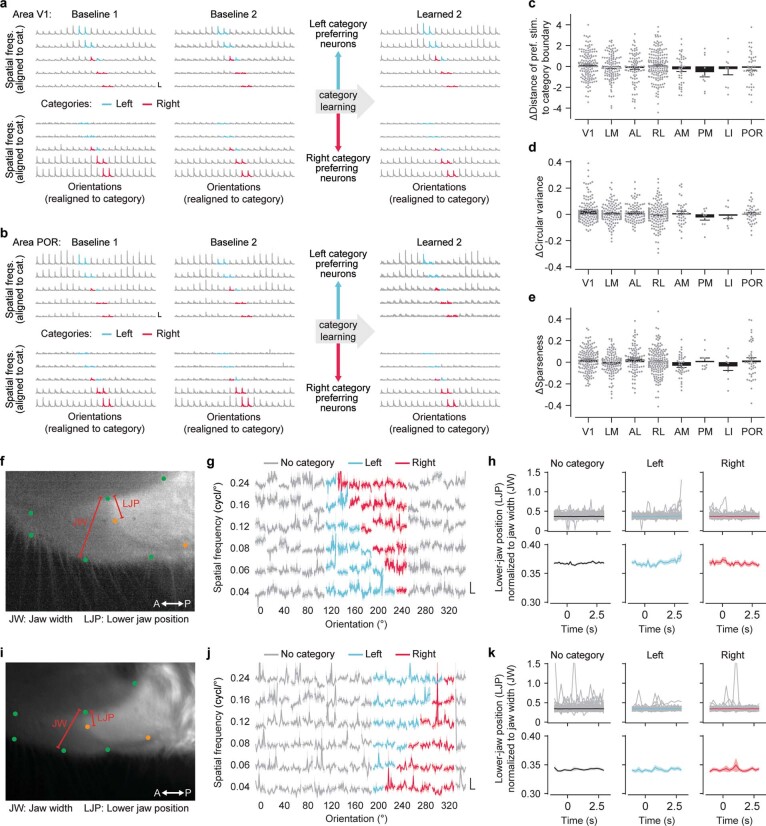
Tuning curves and operant motor responses measured out-of-task. **a**, Top row: Stimulus-aligned inferred spiking activity for all three out-of-task time points, averaged across all V1 neurons that were significantly modulated by visual stimuli in the GLM analysis at in-task time points ‘baseline 3’ and ‘learned 1’ (‘stable’ neurons; Fig. 5) and showed preferential responses to left category stimuli. Bottom row, as top row, for ‘stable’ neurons preferring right category stimuli. Orientation/spatial frequency grids were flipped and shifted such that categories mapped onto the same grid positions. Blue, left category; pink, right category. Scale bars, vertical, 0.5 inferred spikes/s, horizontal, 3 s. **b**, As (**a**), for area POR. **c**, Per area, the differential (‘learned 2’ minus the average of ‘baseline 1’ and ‘baseline 2’) Euclidean distance of the preferred stimulus to the category boundary (calculated using equal weighted steps for orientation and spatial frequency) for ‘stable’ neurons. Bars, mean (±s.e.m.; *n*=635 neurons from 10 mice). **d**, As (**c**), for circular variance^117^ of the orientation tuning curves (*n*=635 neurons from 10 mice). **e**, As (**c**), for sparseness^118^ of the two-dimensional orientation/spatial frequency tuning curves (*n*=635 neurons from 10 mice). **f**, Example video frame showing the mouth of a mouse. Annotations were made using DeepLabCut^109,110^. Green, upper jaw; orange, lower jaw. Annotations defined the position of the lower jaw relative to the upper jaw (LJP) and the width of the upper jaw (JW). **g**, Stimulus-aligned relative opening of the mouth (quantified as the LJP/JW). Stimuli were organized by orientation (horizontal) and spatial frequency (vertical), left and right category stimuli are shown in blue and pink. Scale bars, vertical, 0.05 LJP/JW, horizontal, 2 s. **h**, Relative opening of the mouth (LJP/JW) for stimuli that were not part of a category (left), part of the left category (middle) and part of the right category (right). Upper row, individual stimulus presentations (gray) and their mean (black or color). Lower row, mean (±s.e.m.) across stimulus presentations. Stimulus onset was at 0 s, and lasted 2.5 s. **i-k**, As (**f-h**), for a second mouse.

We observed that, overall, semantic CTI increased after learning (pooled across all ‘stable’ visually modulated neurons; Fig. 6b and Extended Data Fig. 9a). This increase in semantic CTI was most pronounced in areas V1 and POR (Fig. 6c). However, the feature CTI also generally increased after learning (pooled across all ‘stable’ visually modulated neurons; Fig. 6d and Extended Data Fig. 9b), although no individual area stood out specifically (Fig. 6e). We quantified a neuron’s unequivocal tuning for learned categories by subtracting the feature CTI from the semantic CTI, obtaining a single value that reflects whether a neuron’s tuning is better explained by categories or by stimulus features (ΔCTI). Pooled across all neurons, ΔCTI did not change after learning. However, specifically neurons in area POR showed increased ΔCTI values after category learning (Fig. 6f and Extended Data Fig. 9c). This change in category tuning was restricted to in-task recordings, as out-of-task tuning curve measurements showed no difference between baseline and after learning (Extended Data Fig. 10). In summary, only in area POR, did neurons become overall better tuned to categories in comparison to their tuning for orientation and spatial frequency.

A recently developed model of category learning predicts that category selectivity emerges as a consequence of a neuron’s choice probability, the co-fluctuation of activity with behavioral choice^16^. To test whether our data support this idea, we calculated selectivity for behavioral choice from the choice-related component in the GLM, at the imaging time point when mice had successfully learned stimulus discrimination, but had not yet learned to discriminate categories (Fig. 6g). This measure of choice selectivity was significantly correlated with the later quantification of ΔCTI of the same neurons in the in-task category learning time point (Fig. 6h). Specifically in area POR, where we observed an overall increase in ΔCTI, choice selectivity of individual neurons that increased in ΔCTI after learning was, already before category learning, larger than that of neurons that decreased in ΔCTI (Fig. 6i). This suggests that an increased ΔCTI, and thus tuning for semantic rather than perceptual aspects of the categories, in area POR is facilitated by choice selectivity before category learning.

## Discussion

Using a behavioral paradigm for information-integration category learning, we established that mice can perform such a task, discriminating and generalizing stimuli, typically showing a rule bias. Learned visual categorization relied in part on neurons with small receptive fields and could not be performed without visual cortical activity, but did not critically depend on a single visual area. We identified a broad distinction between dorsal and ventral stream areas, with dorsal stream areas responding more universally to visual stimuli, while in ventral stream areas, neurons are more flexibly recruited to respond during visual stimulus presentation after learning. Newly responsive neurons across areas were likely to be selective for behavioral choice and reward. Finally, we identified area POR as the first visual processing stage at which neurons became more tuned to a category boundary, independent from their change in orientation or spatial frequency tuning.

### Implicit versus explicit categorization

Besides a hierarchical distinction in the degree of category selectivity across brain areas, it has been proposed that the brain uses parallel, distinct neural circuits for solving explicit and implicit categorization problems^50^. Explicit categories are often defined by a single rule, making them easily verbalizable. Implicit categories are more procedural in nature, learned by trial and error, require more training and do not necessarily depend on declarative memory^51,52^. Information-integration categories are a specific example of these^24,53^. Based on fMRI studies, explicit, rule-based categories are thought to depend more on activity in frontal areas of the neocortex^54,55^, while implicit categorization relies more on a distributed set of brain regions^6,56^, including the basal ganglia^55^ and possibly sensory cortex^57^. This idea is supported by a human behavioral study showing that rule-based categorization—in contrast to information-integration categorization—does not depend on retinotopic stimulus position^23^. Hence, observing an effect of category learning in retinotopically organized visual areas of the neocortex may be specifically tied to having trained mice on information-integration categories.

Still, to what degree neural systems for explicit and implicit categorization are truly segregated is debated and could for instance depend on perceptual demand and task design^21,58^. It is, for example, also possible that the involvement of sensory areas in our and other studies depends on the particular perceptual demand of information-integration categories and is not caused by its implicit nature. In addition, the reduced category space that we implemented in our chronic imaging experiment often resulted in a strong rule bias. When inspecting individually learned category boundaries in this experiment (Extended Data Fig. 4b), it can be argued that here mice learned, at least to some extent, rule-based categories. Therefore, it would be premature to conclude that the involvement of mouse visual areas in category learning is specific to information-integration categories.

### Ventral and dorsal stream areas are differently modulated by learning

One of our main findings is that, after learning, a subset of recordings showed an increased fraction of neurons significantly driven by in-task visual stimulus presentation. These recordings came predominantly from areas that, in the mouse^41,42^, display a connectivity pattern resembling that of ventral stream areas in higher mammals^43,59,60^. In mice, ventral visual stream neurons have been shown to preferentially tune to slowly moving stimuli, and they have higher spatial frequency preferences in comparison to neurons in dorsal stream areas^36,37,48^. These observations are thought to parallel enhanced tuning for features of complex objects, as observed in monkey temporal cortex^38,61^. Still, the type of features and complexity of visual stimuli that neurons in human and monkey temporal cortex are tuned to^62,63^ do not directly compare to what has been observed in rodents (for example, in ref. ^64^). Beyond hierarchical differences in preferential processing of stimulus features, fMRI experiments have indicated that areas early in the human ventral visual stream can be modulated by learning^21,22,65^. Our study, showing that mouse higher visual areas are differentially modulated by visual learning, thus extends the already existing parallel in functional organization of the visual system of lower and higher mammals.

### An early signature of a semantic representation

The overall aim of our study was to provide a better understanding of how far the trace of a semantic memory extends to sensory regions of the brain. Using category learning^6,66,67^ with well-controlled, simple visual stimuli should, in principle, allow a category representation to form at the very first stages of visual information processing where neurons respond selectively to such stimuli^17,18^, unless there are fundamental limitations in cortical plasticity preventing this. We found that the learned category association depended, in part, on the retinotopic position of presented stimuli, suggesting that the category representation is partly carried by neurons having defined receptive fields in visual space. The approach of disentangling category tuning from feature tuning revealed that, indeed, neurons across all areas of the visual cortex updated their tuning curves for orientation and spatial frequency, as well as for category, such that they could support improved differentiation of the trained categories.

However, in visual area POR, neurons became better tuned to categories than could be explained by their orientation and spatial frequency-specific tuning. These neurons tended to be choice selective already before category learning had started, and the degree of choice selectivity covaried with the amount of category selectivity that was achieved after learning. This is in line with a recent model showing how choice selectivity can drive the tuning of a neuron to change from being orientation-tuned to becoming category-selective^16^. Electrophysiological and imaging experiments have shown that rodent area POR features diverse neural correlates of visual stimuli, behavioral choice, reward and motivational state^68,69^. This diversity could result from POR’s extended network of anatomical connectivity, for example, with lateral higher visual areas^42^, receiving visual drive from the superior colliculus via the lateral posterior nucleus of the thalamus^70^, and reciprocally connecting to the lateral amygdala^71^, the perirhinal and lateral entorhinal cortex^42,72^ and orbitofrontal and medial prefrontal cortex^73^. Possibly, the presence of the various functional correlates, as well as the anatomical connectivity pattern placing it early within the hierarchy of the mouse ventral visual stream^42^, allows for category-selective plasticity to occur and set POR apart from other visual areas.

Thus, it appears that plasticity in eight of the nine recorded visual areas was limited to neurons predominantly shifting their feature tuning in support of categorization, even though we observed many choice-modulated neurons in all local networks (Fig. 5g), suggesting that choice selectivity alone does not explain category tuning. Could it be that plasticity in the eight non-category representing areas is bound by some factor, other than the proposed choice correlation (see above), limiting the speed and range of tuning curve changes? While vastly speculative, one possible mechanism explaining this could be a difference in how broadly neurons in these areas sample their functional inputs, either locally^74^ or long range. If a neuron in, for example, area LM would have more like-to-like connectivity compared to a POR neuron, the LM neuron would be more strongly bound to its functional properties compared to the POR neuron. Future work on local and inter-area functional and anatomical connectivity might reveal such differences in connectivity motifs.

In summary, we find that area POR has a neural representation that increases in size after learning, and is biased to reflect categories (that is, semantic information), rather than orientation and spatial frequency tuning (that is, perceptual information). We propose that this elementary category representation propagates from area POR, via parahippocampal regions and basal ganglia, to (pre)frontal cortex^75^, there forming a highly selective and context-specific learned category representation^12,76^. Thus, the representation of semantic information emerges early—albeit not at the first processing stage—in the ventral stream of the mouse visual system.

## Methods

### Mice

All experimental procedures were conducted according to institutional guidelines of the Max Planck Society and the regulations of the local government ethical committee (Beratende Ethikkommission nach §15 Tierschutzgesetz, Regierung von Oberbayern). Adult male C57BL/6 mice ranging from 6 to 10 weeks of age at the start of the experiment were housed individually or in groups in large cages (type III and GM900, Tecniplast) containing bedding, nesting material and two or three pieces of enrichment such as a tunnel, a triangular-shaped house and a running wheel (Plexx). In a subset of experiments (stimulus-shift experiment, *n* = 3; local cortical inactivation experiment, *n* = 3), we used mice (12 to 15 weeks old; two female and one male) that expressed the genetically encoded calcium indicator GCaMP6s in excitatory neurons (B6;DBA-Tg(tetO-GCaMP6s)2Niell/J (Jax, 024742) crossed with B6.Cg-Tg(Camk2a-tTA)1Mmay/DboJ (Jax, 007004))^77,78^. All mice were housed in a room having a 12-h reversed day/night cycle, with lights on at 22:00 and lights off at 10:00 in winter time (23:00 and 11:00 in summer time), a room temperature of ~22 °C and a humidity of ~55%. Standard chow and water were available ad libitum except during the period spanning behavioral training, in which access to either food or water was restricted (for a detailed procedure see ref. ^79^).

### Head bar implantation and virus injection

A head bar was implanted under surgical anesthesia (0.05 mg per kg body weight fentanyl, 5.0 mg per kg body weight midazolam, 0.5 mg per kg body weight medetomidine in saline, injected intraperitoneally) and analgesia (5.0 mg per kg body weight carprofen, injected subcutaneously (s.c.); 0.2 mg ml^−1^ lidocaine, applied topically) using procedures described earlier^79^. Next, a circular craniotomy with a diameter of 5.5 mm was performed over the visual cortex and surrounding higher visual areas. The location and extent of V1 was determined using IOS imaging^37,80,81^ and the locations of higher visual areas were extrapolated based on the acquired retinotopic maps and literature^36,37,82,83^. A bolus of 150 nl to 250 nl of AAV2/1-hSyn-GCaMP6m-GCG-P2A-mRuby2-WPRE-SV40 (ref. ^84^) was injected at 50 nl min^−1^ in the center of V1 and into four to six higher visual areas at a depth of 350 μm below the dura (viral titers were 1.24 × 10^13^ and 1.02 × 10^13^ GC per ml). Following virus injection, the craniotomy was closed using a cover glass with a diameter of 5.0 mm (no. 1 thickness) and sealed with cyanoacrylate glue and a thin edge of dental cement. Animals recovered from surgery under a heat lamp and received a mixture of antagonists (1.2 mg per kg body weight naloxone, 0.5 mg per kg body weight flumazenil and 2.5 mg per kg body weight atipamezole in saline, injected s.c.). Postoperative analgesia (5.0 mg per kg body weight carprofen, injected s.c.) was given on the next 2 d. In some animals, we performed a second surgery (following procedures as described above) to remove small patches of bone growth underneath the window.

### Visual stimuli for information-integration categorization

Visual information-integration categories were constructed from a 2D stimulus space of orientations and spatial frequencies, in which the category boundary was determined by a 45° diagonal line^6,53,58,85^. In experiments with freely moving mice, the category space consisted of stationary square-wave gratings of approximately 7 cm in diameter, having one of seven orientations equally spaced by 15° between the cardinal axes, and seven spatial frequencies (0.03, 0.035, 0.04, 0.05, 0.07, 0.09 and 0.11 cycles per degree, as seen from a distance of 2.5 cm). Stimuli exactly on the diagonal category boundary were left out, resulting in two categories with 21 stimuli each (Fig. 1b). In the touch screen task, animals tended to weigh orientation over spatial frequency, which is possibly the result of greater variability in perceived spatial frequency than orientation during the approach to the screen.

In experiments with head-fixed mice, visual stimuli consisted of sinusoidal drifting gratings presented in a 32° diameter patch and extended by 4° wide faded edges, on a gray background. The stimulus was positioned in front of the mouse with its center at 26° azimuth and 10° elevation. In experiments without chronic imaging, stimuli had one of seven orientations spaced by 20°, and one of six spatial frequencies (0.04, 0.06, 0.08, 0.12, 0.16 and 0.24 cycles per degree) and drifted with 1.5 cycles per degree in a single direction. The category space was always centered on one of the cardinal orientations (for example, centered on 180° resulted in a stimulus range from 120° to 240°). The category boundary had an angle of 45° and was placed such that no stimuli were directly on the boundary (Fig. 2b). In these experiments, animals tended to weigh spatial frequency more strongly than orientation, which could indicate that the differences in spatial frequency were perceived as more salient.

For experiments in which the stimulus position was altered, the center of the computer monitor was repositioned from the default setting (right of the mouse, 26° azimuth) to a position straight in front of the mouse (0° azimuth) or left of the mouse (−26° azimuth; Fig. 2c). The monitor rotated on a swivel arm that was secured below the mouse such that the foot point (the point closest to the eye) was always in the center of the monitor. In addition, we verified that at each position the monitor was equidistant to the mouse. The relative position of the stimulus on the computer monitor and all other features were kept constant.

For chronic imaging, most stimulus parameters were identical to experiments without imaging. The complete stimulus space consisted of a full 360° range of orientations spaced by 18° (two directions of motion per orientation) and five spatial frequencies (either 0.06, 0.08, 0.12, 0.16 and 0.24 cycles per degree or 0.04, 0.06, 0.08, 0.12 and 0.16 cycles per degree). For each mouse, the category space was selected to contain six consecutive orientations (spaced by 18°) and the full range of five spatial frequencies, centered on one of the cardinal orientations (for example, centered on 180° resulted in a range from 135° to 215°). However, the stimuli were reduced in number; only the stimuli furthest from the boundary (initial stimuli) and closest to the category boundary (category stimuli) were used in the behavioral task (Fig. 3g). The reduced category space was implemented to consist of fewer stimuli, such that each stimulus would have a larger number of presentations (trials), thus facilitating a precise assessment of stimulus and category selectivity in the neural data. The angle of the category boundary in chronic imaging experiments was adjusted for rule bias to 23° (or 67° in two mice) to aid the animals that were biased to follow information of a particular stimulus dimension (see Extended Data Fig. 4b for the individual category space of each chronically imaged mouse).

### Touch screen operant chamber

Conditioning of freely moving animals was done in a modular touch screen operant chamber (MED Associates), which was operated using commercial software (K-LIMBIC) and was placed in a sound-attenuating enclosure^86–88^. The north wall of the operant chamber consisted of a touch screen with two apertures in which visual stimuli were presented, and a small petri dish that served as receptacle for a food pellet (equivalent to regular chow; TestDiet 5TUM). The south wall housed a lamp, a speaker and a retractable lever, and the east wall of the chamber held a water bottle.

Animals were pretrained in three stages. First, food-restricted mice were habituated to the experimental environment for a single, 20-min session, during which they were placed in the operant chamber and in which the food pellet receptacle contained 20–30 food pellets. In the next stage, the animals were exposed to a rudimentary trial sequence. After a 30–60-s intertrial interval, two visual stimuli were presented in the apertures of the touch screen monitor. The stimuli differed in both spatial frequency and orientation. Touching one of the two stimuli (the rewarded stimulus) led to delivery of a food pellet in the receptacle (food tray), while touching the other stimulus had no effect. If the mouse did not touch the rewarded stimulus within ~30 s from stimulus onset, the trial timed out and the next intertrial interval started. This stage lasted for two to four daily sessions (each lasting 1–1.5 h), until the mouse performed at least 50 rewarded trials. In the final pretraining stage, the lever was introduced. The trial sequence was almost identical to the previous stage, except now the trial started with lever extrusion instead of visual stimulus presentation. The visual stimuli were only presented after the mouse had pressed the lever. If the mouse failed to press the lever within ~30 s, the trial timed out (without visual stimulus presentation) and the sequence proceeded with the next intertrial interval.

Mice switched to the operant training paradigm as soon as they performed over 50 rewarded trials in the last pretraining stage. The trial sequence was very similar to the pretraining sequence, a 30–60-s intertrial interval was followed by lever extrusion (Extended Data Fig. 1b). When the mouse pressed the lever, it was retracted, and two visual stimuli were presented in the apertures of the touch screen. One stimulus was selected from the rewarded category and one stimulus was selected from the non-rewarded category such that they mirrored each other’s position across the center of the category space. If the mouse touched the screen within the aperture where the rewarded stimulus was presented, a food pellet was delivered in the receptacle. If the mouse touched the non-rewarded stimulus, the trial ended and proceeded to the next intertrial interval. Because the intertrial interval already lasted 30–60 s, no additional time-out or other punishment was implemented.

Finally, after mice had learned discriminating the first set of two stimuli (>70% correct), we introduced four additional stimuli, one step closer to the category boundary. The original stimuli were also kept in the stimulus set. If there was a reduction in performance, animals were trained for a second day on this new stimulus set. Over the next 3 d, we introduced six, eight and ten additional stimuli. The set of ten stimuli was trained for 2–3 d, after which we added the final 12 stimuli and the animals discriminated the full information-integration categorization space (Fig. 1b).

### Head-fixed operant conditioning

Head-restrained conditioning was performed in a setup described in ref. ^79^. In brief, the mouse was placed with its head fixed, on an air-suspended Styrofoam ball^89,90^, facing a computer monitor (Fig. 2a). The computer monitor was placed with its center at 26° azimuth and 0° elevation. The monitor extended 118° horizontally and 86° vertically, and pixel positions were adapted to curvature-corrected coordinates^37^. Two lick spouts were positioned in front of the mouse within reach of the tongue^91^. The setup recorded licks on each spout, as well as the running speed on the Styrofoam ball using circuits described in ref. ^79^. Water rewards were delivered through each lick spout by gravitational flow using a fully opening pinch valve (NResearch). The setup was controlled by a closed-loop MATLAB routine using Psychophysics Toolbox extensions^92^ for showing visual stimuli, and in addition, all signals were continuously recorded using a custom-written LabView routine.

Before head-fixed training, animals were habituated by handling, exposure to the Styrofoam ball and by drinking water from a handheld lick spout. After the habituation period, animals underwent head-fixed pretraining in two stages.

Pretraining phase 1 consisted of trials in which animals were trained to lick for reward on a single lick spout. Each trial in this training phase started with an intertrial interval of 2 s, followed by a period during which the mouse had to withhold from running and licking for 0.5 ± 0.05 s (a no-lick, no-run period). Next, stimulus presentation commenced, with the stimulus randomly selected from the full set of stimuli (all combinations of five different spatial frequencies and ten different orientations, moving in two directions; ‘Visual stimuli for information-integration categorization’). Stimulus presentation lasted 0.9 ± 0.1 s. After stimulus presentation, and a 0.1-s delay, there was a period in which the mouse could make a response (response window), lasting 10 s. The first lick on the lick spout within the response window resulted in immediate delivery of a water reward. The trial would count as correct and the trial sequence proceeded into the intertrial interval of the next trial. If the mouse did not make a lick, the response window would time out, the trial counted as a miss and the trial sequence also proceeded into the intertrial interval. The goal of this stage was to familiarize the mouse with the general sequence of withholding licking and running, stimulus presentation and licking for reward. Animals were typically kept in this stage for 4–6 d, and during these days the intertrial interval was gradually lengthened to 5 s.

Pretraining phase 2 consisted of the same basic trial structure as phase 1, but had two available lick spouts. During phase 2, the no-lick, no-run period was increased to 0.7 ± 0.1 s, stimulus presentation was lengthened to 1.5 ± 0.1 s and the delay between stimulus offset and response window was increased to 0.2 ± 0.1 s. The presented stimulus was chosen randomly from the same set as in phase 1, but now only one of the two lick spouts was randomly assigned for reward delivery (there was no relation between the stimulus and the rewarded lick spout). Water reward was given after the mouse had licked the predetermined lick spout. If the mouse licked the other spout, it had no effect on the trial flow; that is, the mouse could still lick the other spout and obtain the reward within the period of the response window. Pretraining phase 2 lasted until the animal performed >50 trials per day, and at least until the period of out-of-task baseline imaging ended (duration ranging between 7 and 17 d).

Following pretraining, animals were initially trained using two stimuli, one requiring a lick response on the left lick spout and one on the right lick spout. These training sessions implemented the same trial structure as pretraining phase 2 (Extended Data Fig. 2b), but now the stimuli indicated the side of the lick spout that would give a drop. For the first three to five training sessions, licks on the incorrect spout did not alter the trial flow (these sessions are marked as ‘shaping’ in the timeline in Extended Data Fig. 2a). After these initial shaping sessions, a lick on the incorrect spout during the response window period resulted in a time-out stimulus (black bar, 8° high and 106° wide, centered on the computer monitor), which was presented for the duration of the 2-s time-out. Time-out stimuli were not shown during imaging. After initial stimuli were discriminated with more than 70% correct, we gradually introduced more stimuli for categorization. As long as performance stayed above 65% correct trials, we added stimuli that were, each time, one step closer to the category boundary until the full categories were discriminated.

During pretraining phase 2 and subsequent training, an automated lick-side bias-correction algorithm directed the setup to increase the number of trials having the active lick spout on the side that the animal did not prefer (see ref. ^79^). This algorithm was stopped as soon as the animal showed signs of above-chance stimulus discrimination and was never implemented during sessions in which imaging was performed during the behavioral task. In a subset of experiments, we initially displaced the retinotopic position of the stimuli to the left and the right sides of the monitor (−16 and +16° azimuth) in such a way that it matched the side of the active lick spout where the response should be made. This was done to facilitate learning of the ‘lick-left’/‘lick-right’ association. This training stage is marked ‘shifted’ in the timeline depicted in Extended Data Fig. 2a. After mice reached the criterion using this position-shifted paradigm, we gradually shifted all stimuli to the center position and proceeded with the imaging of the time point ‘stimulus discrimination’ only when stable high performance was maintained without stimulus shifts.

In a subset of experiments (three of five mice from the experiment in which the monitor position was altered and in experiments presented in Extended Data Fig. 5), we connected the above-described lick-side bias-correction algorithm to a servo system that could micro-adjust the left/right position of the lick spouts. While online adjustments of the lick spout position were not made often, this method of physically opposing the lick spout position to the side bias could correct the left/right licking behavior of mice that occasionally defaulted to respond only on a single lick spout. These online adjustments, however, could not in any way affect behavioral performance or category-specific choices of the mouse.

### Time points of image acquisition

Imaging sessions were performed throughout the experiment and differed in several aspects. Each imaging time point was acquired over multiple days, with a different visual cortical area imaged on each day. For each mouse, the same subset of cortical areas was imaged at every imaging time point throughout the experiment. Thus, each time point contained the same complete cycle through all areas (Fig. 3a). We acquired imaging data using two different visual stimulation protocols, one for in-task imaging and one for out-of-task imaging.

Out-of-task imaging sessions were acquired at two baseline imaging time points, during the period of pretraining. In addition, one out-of-task time point was acquired at the end of the chronic imaging experiment (Fig. 3a). Out-of-task imaging sessions were always acquired after the behavioral session had been completed, thus the animal was in a satiated state. In these imaging sessions, the setup was kept in the same configuration as during behavioral training, except for that the lick spouts were moved out of the mouse’s view. The imaging sessions started with 15 min of darkness, followed by ~15 s of gray screen (50% luminance, allowing the animals to adapt to the screen brightness). Next, stimuli were presented, interleaved by periods of a gray screen. The stimuli were presented in eight blocks containing all 100 unique stimuli (all combinations of ten orientations, moving in two directions and five spatial frequencies). The order of stimulus presentation was shuffled within each block individually.

In-task imaging sessions started with 12 min of darkness, followed by ~15 s of gray screen (50% luminance) during which the mouse usually received a few drops of water to indicate that the task was about to start. After this pre-task period, the visual categorization task started and lasted for 35 min. At the end of the imaging session, there was another period of 12 min darkness and a 3-min period in which a water reward was given roughly every 20 s, on either the left or the right lick spout (pseudorandom side assignment per reward). In-task imaging sessions were performed at three distinct time points (Fig. 3a). The first in-task imaging time point was acquired during the baseline period, directly after pretraining was finished. At these time points, both the initial and the category stimuli were included in the stimulus set. If the animal made a mistake in such a session (that is, a lick on the incorrect side), no punishment or time-out was implemented (that is, the animal could still obtain a reward by making a lick on the other spout). In three animals, we performed a full repeat of the in-task baseline imaging time point. The second in-task imaging time point was acquired after the animal had reached the criterion on the visual discrimination task. At this time, only the initial stimuli were shown. Incorrect choices were always followed by a time-out, but without the visual time-out stimulus being shown. The final in-task imaging time point was acquired after the animal performed above chance on the category learning task. This task included only the category stimuli.

### Muscimol inactivation

At the end of the chronic imaging time series, five mice underwent two experiments on consecutive days, in which visual cortical areas were inactivated, or a control manipulation was performed. The order of cortical inactivation and the control experiment was counterbalanced across mice. Under isoflurane anesthesia (3% induction and 1.5% maintenance in O_2_), the chronically implanted cranial window was opened and the surface of the exposed cortex was treated for 20 min with a solution containing 5 mM muscimol in aCSF^93^. Subsequently, the cortex was covered with 0.75% agarose (in aCSF) containing 5 mM muscimol, and sealed with a cover glass. The mouse was allowed to recover for approximately an hour. During the behavioral experiment following this manipulation, we performed calcium imaging of L2/3 and L5 neurons in primary visual cortex to confirm cortical inactivation. The control experiment was executed in the exact same way, except that muscimol was not added to the aCSF.

For the targeted inactivation of specific visual cortical areas, three mice that were extensively trained on the information-integration category task underwent a series of muscimol (inactivation) and saline (control) injections into retinotopically determined visual cortical areas (V1, AL and POR). In all mice, inactivation and control conditions were interleaved by one day of behavioral training without manipulation (for timeline, see Extended Data Fig. 5a). Mice were lightly anesthetized with isoflurane (3% for induction and 1.2–1.5% for maintenance in O_2_), the chronically implanted window was opened and either a 25-nl solution of 5 mM muscimol in saline or 25 nl saline was injected 300 µm below the cortical surface. The injection parameters were calibrated to result in a spread of the injected solution approximately 700 µm radially from the injection center (Extended Data Fig. 5b). Injections targeted at area AL were done slightly more anterolaterally such that they likely also affected area RL, but not area LM. Injections targeted at area POR likely inactivated areas LI and LM also. Following the injection, the cortex was sealed with a cover glass. After approximately an hour of recovery, categorization behavior was tested.

### Intrinsic signal imaging

IOS imaging was performed according to methodology described before^80^. For IOS imaging during window implantation surgery, we illuminated the exposed, cleaned skull, within the 7-mm-diameter central opening of the head bar. We centered an approximately 5 × 5-mm FOV on stereotaxic coordinates of V1 and focused the image on the surface of the exposed skull using green light (540 nm). For IOS imaging through an implanted cranial window, we centered the FOV on the window and focused the image on the dural and pial blood-vessel pattern. Next, we changed the illumination wavelength to 740 nm (emission filter of 740 nm, full-width half-maximum value of 10 nm) and moved the focal plane down to approximately 800 μm below the skull surface, which was an estimated 300–400 μm below the pial surface. Images were acquired using a Teledyne DALSA Dalstar CCD camera and a Matrox frame grabber. Data processing and storage were done using a custom-written image acquisition and analysis program in MATLAB (MathWorks). During the period of image acquisition, we presented visual stimuli on a curvature-corrected^37^, gamma-corrected, LCD monitor (DELL; 59.9 cm wide and 33.8 cm high). The monitor background luminance was kept at 50% gray values, which was equiluminant to the visual stimuli when averaged over a larger area.

For discrete retinotopic maps^81^, the visual stimulus was a square-wave grating (0.04 cycles per degree), drifting at two cycles per second in eight directions in a semi-random sequence (500 ms per direction). The stimulus was presented for a duration of 6 s in a square or rectangular aperture of a specific retinotopic size (that depended on the number of apertures (patches) used for mapping). We typically used four or six patches for IOS imaging during window implantation surgery, thus presented the stimuli in a 2 × 2 or 2 × 3 vertical/horizonal grid. When imaging through an already-implanted cranial window, we typically used 12 (3 × 4), 15 (3 × 5) or 24 (4 × 6) patches. Stimulus presentations were interleaved by a 12-s inter-stimulus interval.

For continuous retinotopic maps^37,94^, we presented a checkerboard stimulus in a wide rectangular aperture spanning 20° on one axis and the full width/height of the monitor on the other axis. The checkerboard pattern consisted of a grid of full-contrast black and white patches, ~12° in size, repositioned and contrast inverted every 166 ms. The aperture in which the checkerboard was displayed drifted continuously across the screen. Each of the four cardinal drift directions looped either 10–20 times at a drift speed of 3–4° per second or 40–50 times at a drift speed of 15–20° per second, with a 30-s pause in between sets of drift-direction loops.

### Two-photon calcium imaging

In vivo two-photon calcium imaging^95^ was performed with a customized commercially available Bergamo II (Thorlabs) two-photon laser scanning microscope^96^ using a pulsed femtosecond Ti:Sapphire laser (Mai Tai HP Deep See, Spectra-Physics) and controlled by ScanImage 4 (ref. ^97^). The calcium indicator GCaMP6m^98^ and the structural marker mRuby2 (ref. ^99^) were both excited with a wavelength of 940 nm. Emitted photons were filtered for reflected laser light (720/25 short-pass filter), spectrally separated using a dichroic beamsplitter (FF560) and two band-pass filters (500–550 nm for GCaMP6m; 572–642 nm for mRuby2) and detected using two GaAsP photomultiplier tubes. Laser power was kept between 18 and 35 mW, depending on the depth of imaging and the quality of the chronic window. Images were acquired from two alternating planes, 40 μm apart, using a ×16 0.8-NA objective (Nikon) mounted on a piezoelectric stepper (Physik Instrumente). The *xy* image dimensions were 325 × 250 μm (512 × 512 pixels), and each image plane was acquired at a rate of ~15 Hz (total frame rate of ~30 Hz).

### Image processing

The background signal of the photomultiplier tubes was measured at the start of each imaging stack, and the mean background signal level was subtracted from the entire stack (dark noise subtraction). Lines in the images were scanned bidirectionally and an inadvertent line shift was corrected for by calculating the maximum cross-correlation of lines scanned in each direction. Image planes from acquired stacks were realigned to correct for in-plane movement artifacts, using an algorithm that calculates the maximum cross-correlation of the Fourier transforms of two images^100^.

### Within-session and across-session region of interest identification

To assist with image annotation, we produced a high signal-to-noise average image for each channel from the resulting stack as well as a maximum projection image using a running average of 5 s. In addition, we calculated a Δ*F/F* stimulus locked-response image in which brightness of the pixels indicated the stimulus-induced increase in fluorescence relative to baseline, for that pixel. The outlines of neuronal regions of interest (ROIs) from five mice were annotated manually by using the average image of each channel, but with assistance of the maximum projection and the Δ*F/F* response image. Annotations were made by one of three experimenters, and subsequently adjusted by a single experimenter using a custom-written MATLAB (MathWorks) program.

These manually annotated image stacks were used to train two multilayered convolutional neural networks programmed using Tensorflow^101^ and Python3, which were then used to annotate the imaging stacks for five additional mice (https://github.com/pgoltstein/NeuralNetImageAnnotation/). One network annotated the centers of neurons (5 × 5-pixel centroid region) and the other annotated the complete somata of neurons on a pixel-by-pixel basis. We used the average image of both imaging channels, as well as the Δ*F/F* response image as source data for the annotation. The input layer of the network supplied a 33 × 33-pixel FOV around each single pixel, thus its dimensions were 33 × 33 pixels by three channels. The network had four 3 × 3 convolutional layers with 2 × 2 max-pooling applied to each of these layers, and 16, 32, 64 and 128 channels in each layer, respectively. The last convolutional layer was connected to a fully connected layer containing 512 units, and the fully connected layer in turn connected to two output layer units, one indicating that the pixel was part of the ROI center or body, and one unit indicating the inverse. All layers consisted solely of rectifying linear units.

The network was trained by minimizing the softmax cross-entropy using the Adam optimizer^102^ on repeated batches of 2,000 samples, drawn equally from the training data (512 × 512 pixels from 122 images from five mice). Regularization during training was implemented by dropout in the fully connected layer with a probability of 0.5. Each network was trained using a learning rate varying between 10 × 10^−3^ and 10 × 10^−5^. The centroid-detecting network was trained on 10.6 × 10^6^ samples, and the cell-body-detecting network was trained on 96.1 × 10^6^ samples. Cross-validated pixel-wise performance was determined using 122 different annotated images of the same mice. The centroid-detecting network performed at 87.5% correct (precision of 0.95, recall of 0.79) and the cell-body-detecting network performed at 86.6% correct (precision of 0.88, recall of 0.84). Next, an algorithm identified centers of individual cells from the network-centroid annotations and used the network-body annotations to detect the outlines of these cells. Network annotations were further corrected by a single experimenter using a custom-written MATLAB(MathWorks) program.

Before further processing, we removed overlap between annotations using an algorithm. In addition, we removed all (parts of) annotations that, due to motion artifacts, shifted out of the FOV for more than 0.1% of the stack. We aligned annotations of all stacks from a single chronic recording using a custom-written MATLAB (MathWorks) program that matched ROIs across imaging sessions using an affine transform and allowed additional manual control over alignment parameters. Neurons that shared more than 50% overlap of the cell-body pixels were defined as a putative matched group. Finally, we manually inspected and corrected all matched groups that were present in all chronic recordings for continuity, missed annotations or false-positive annotations.

### Neuronal region of interest signal extraction

For each ROI, we calculated a GCaMP6m and mRuby2 fluorescence signal by taking the mean of all pixels within the ROI, for each channel separately. In addition, we calculated a local neuropil signal, a measure of local fluorescence intensity, over a circular region surrounding the ROI (2–33-μm ring). Using these signals, we first compensated for non-cell-specific fluorescence bleeding into the ROI signals by subtracting the neuropil signal time series, multiplied by 0.7 from the raw fluorescence time series, a method known as neuropil correction^98,103,104^. The median of the neuropil time series (multiplied by 0.7) was added, to offset the lower baseline fluorescence signals resulting from neuropil correction. Next, we compensated for small fluctuations in fluorescence that followed changes in the axial position of cells (for example, due to slow drift or motion artifacts) by calculating the ratio (*R*) between the green and red channel, as both channels should be affected equally by such out-of-plane motion^105^.

For each frame, an *R*_0_ value was calculated from the lowest 25% values in a 60-s window around that frame. The Δ*R/R* value was calculated by subtracting the *R*_0_ value from the fluorescence value (*R*) of a frame and dividing the remainder over the *R*_0_ value (adapted from ref. ^106^). To further remove artifacts, the resulting GCaMP6m Δ*R/R* fluorescence time series was processed using the constrained FOOPSI algorithm^107,108^, which fits the calcium Δ*R/R* time series with a biologically plausible model and provides an inferred spike time series for each neuron with high temporal resolution that was used in all following analyses. Visualized traces of inferred spike activity were smoothed with a five-frame flat kernel.

### Analysis of behavioral data

Behavioral performance was reported as the fraction of correct trials. In the touch screen task, this was quantified as the number of trials in which the mouse touched the correct (rewarded) stimulus, divided by the total number of trials in which the mouse made a touch response. In the ‘lick-left’/’lick-right’ task (head-fixed), this was quantified as the number of trials in which the animal licked on the correct lick spout, divided by the total number of trials in which the mouse made a lick response. Steepness of categorization, a function of the distance of stimuli to the category boundary, was determined from the steepness parameter of a fitted sigmoid curve.

While information-integration categories were trained with a systematic boundary requiring the linear integration of the two stimulus features, orientation and spatial frequency, not all mice bisected the stimulus space using the trained boundary angle. The boundary angle, as behaviorally expressed by the animal, was calculated by fitting a 2D plane through a three-dimensional space having orientation and spatial frequency on the *x* and *y* axes, respectively, and performance on the *z* axis. The behaviorally expressed boundary was defined as the intersection of the fitted plane with the plane *z* = 0.5. For category spaces with a reduced number of stimuli (as used in the chronic imaging experiment), the behaviorally expressed boundary vector was calculated using a support vector machine.

During behavioral experiments in which we shifted the stimulus position, we tracked the positions of both eyes using infrared cameras (The Imaging Source). We manually annotated the outlines of the eyes and pupils in a set of sample images using DeepLabCut^109,110^ and used the software to further annotate the movies (see Extended Data Fig. 2g for examples). The pupil diameter was calculated as the average distance between each of four sets of opposing markers on the pupil outline. Horizontal eye position was calculated as the distance from the center of the pupil (the mean of the *x* and *y* coordinates of the eight markers on the pupil outline) to the marker on the left side of the outline of the eye. Both pupil diameter and horizontal eye position were normalized to the width of the eye, defined as the distance between the left and right marker on the outline of the eye. Similarly, during two control experiments, we tracked features of the mouth of the mouse (see example annotated video frames in Extended Data Figs. 10f,i). We quantified the variable ‘relative mouth opening’ as the distance between the central marker on the upper-left jaw and the anterior marker on the lower jaw, normalized to the distance between the central markers on the upper-left and upper-right jaws (Extended Data Figs. 10f,i).

### Image analysis

Discrete retinotopic stimulation was analyzed for each retinotopically specific patch individually. The intrinsic signal response per pixel was quantified as percentage decrease during stimulus presentation (mean signal from 1 s to 6 s after stimulus onset) relative to baseline (mean signal from −6 s to −1 s before stimulus onset). The 2D maps of the IOS response per trial were averaged and smoothed to result in a single average intrinsic signal response map for each retinotopic stimulus position. These average maps were normalized to values of between 0 and 1, to compensate for lower signal strength of patches in the eccentricity of the visual field. From the individual average maps, a single image was constructed by assigning every pixel a color, based on the patch that elicited maximum activity (each retinotopic stimulus position was associated with a unique color).

Periodic visual stimulation was analyzed as described in ref. ^37,94^. In brief, the time series of each pixel in the continuous acquisition was low-pass filtered at four times the slowest stimulus-repetition frequency. The phase and power of the intrinsic signal at the stimulus-repetition frequency were determined for each pixel using a Fourier transform. Retinotopic maps detailing visual-response amplitude and preferred azimuth and elevation were subsequently produced by recalculating the phase to a position in monitor space and scaling the image by the signal power. Finally, equi-elevation and equi-azimuth lines were overlaid on a wide-field image of the cortical blood-vessel pattern.

HLS maps were calculated on a pixel-by-pixel basis from calcium imaging time series. First, a baseline fluorescence map was calculated by averaging all images acquired in the intertrial intervals preceding a visual stimulus presentation. Similarly, a stimulus fluorescence map was calculated for each stimulus individually by averaging all images acquired in the period from visual stimulus onset to 0.5 s after stimulus offset. A Δ*F/F* response map was subsequently calculated by subtracting the baseline from each stimulus map and dividing the remainder over the baseline map. For each pixel, the color (hue) was selected based on the stimulus that gave the largest Δ*F/F* response. The brightness (lightness) of each pixel was determined by the Δ*F/F* response amplitude to the best stimulus. Color intensity (saturation) was determined by calculating the resultant length of the stimulus-averaged Δ*F/F* responses, sorted from largest to smallest, mapped onto a circular space. This resulted in a value of 1.0 when only a single stimulus elicited a response, displaying full color saturation of the pixel. The resultant length was 0.0 when all stimuli drove equal Δ*F/F* response amplitudes, resulting in a white pixel. Multiple HLS maps detailing retinotopic position preference (for example, center versus surround of the visual field) were stitched together using coordinates from the microscope’s motor position controller, to produce a wide FOV HLS map with cellular resolution (Fig. 3b and Extended Data Fig. 3).

### Fraction of responsive neurons

For each imaging session, we quantified the fraction of responsive neurons using inferred spiking activity in the first second of visual stimulus presentation of trials featuring stimuli that were part of the reduced category space (the 1-s period was chosen because it contained relatively few running and licking events, and no rewards occurred). If the recording was an out-of-task imaging time point, in which each stimulus was repeated eight times, we performed a Mann–Whitney *U* test comparing the 1-s period just before stimulus onset to the 1-s period directly after stimulus onset. The following responsiveness criteria were applied for each stimulus: (1) the non-parametric test indicated a significant difference (*P* < 0.05) and (2) the peak inferred spike rate difference was at least 0.01. A neuron was classified as being responsive, when these criteria were met for at least a single stimulus of the reduced category space (containing ten stimuli).

In-task time points were analyzed slightly differently, because the chance of detecting responsive neurons scaled with the variable number of trials that the animals performed. We used subsampling to allow a direct comparison of the fraction of responsive neurons with the out-of-task time points. For each stimulus, we randomly sampled eight trials from the total number of trials, performed the same testing criteria as described above, and repeated the procedure 100 times, resulting in 100 estimates of the stimuli that a neuron was responsive to. From these data, we calculated the probability of the neuron being significantly responsive to at least one stimulus by dividing the number of subsamples with at least one significant stimulus over the total of 100 repeats.

Thus, each method resulted in a single vector listing the probability, per neuron, that it significantly responded to at least one visual stimulus. The out-of-task sessions resulted in binary entries reflecting probabilities of zero and one. The in-task sessions resulted in vectors having values on the interval (0, 1), reflecting probabilities that neurons were significantly responsive on a more continuous scale, as derived from subsampling. By averaging this vector, we obtain in both cases the probability of observing that a randomly chosen neuron from that session is significantly responsive, which, importantly, is equivalent to the overall fraction of responsive neurons per session.

The time-varying patterns of the fraction of responsive neurons across imaging time points (Fig. 4a and Extended Data Fig. 6a) were normalized to the range from 0 to 1, and grouped into clusters using the *k*-means clustering algorithm (scikit-learn). For different values of *k* (2 to 8), we compared the cluster inertia (within-cluster sum of squares) of the actual data to the mean cluster inertia of 100 shuffles (Fig. 4b). The difference between the real and shuffled cluster inertia indicates clustering performance and suggested that the data were best grouped into two clusters (Fig. 4b). Performing the same analysis, but with an artificial third cluster included, accurately detected three clusters. Furthermore, while the *k*-means algorithm has a random initialization step, multiple runs of the same analysis resulted in the same cluster groupings.

Using linear regression, we aimed to identify four components making up the time-varying pattern of fraction responsive neurons (as visually depicted in Fig. 4g). The components were: (1) a stable, non-time-varying fraction (baseline), which was assigned the value 1 at each time point; (2) an exponentially decaying fraction, having the value 1 for the first time point, 0.5 for the second, and so on; (3) a task modulation component having the value 1 for the in-task time points and 0 otherwise; and (4) a learning associated component having a value of 1 for all the post-category learning time points and 0 otherwise. We calculated the contribution of each time-varying component, by applying an NNLS fitting algorithm (SciPy) on the time-varying fractions of responsive neurons of each individual chronic recording. In general, the linear model fitted the time-varying fraction of responsive neurons well (*R*^2 ^= 0.77 ± 0.21 s.d.; *n* = 39 chronic recordings), and each regressor made a unique contribution to the explained variance (decay, Δ*R*^2 ^= 0.254 ± 0.056 (s.e.m.); task, Δ*R*^2 ^= 0.408 ± 0.052 (s.e.m.); learning, Δ*R*^2 ^= 0.095 ± 0.035 (s.e.m.); we did not quantify the unique contribution of base, as it is the intercept and cannot be shuffled; see below for a detailed explanation of Δ*R*^2^).

### Encoding model

To determine how individual task-related and other measured covariates influenced a neuron’s inferred spiking activity, we used a generalized linear model (GLM; encoding model) to predict the inferred spikes of each neuron per imaging frame^15,19,111–113^. Regressors for discrete events such as stimulus onset were represented by a boxcar function, while regressors for continuous parameters such as running speed were represented by scalar values for each imaging frame. All regressors were smoothed with a Gaussian kernel (*σ* = 0.5 s) and repeated over a defined range with 0.5-s steps (see Supplementary Table 3 and Fig. 5a,b for all individual regressors and their ranges).

Stimulus-onset aligned regressors encoded the stimulus parameters: orientation, spatial frequency and trained category. In addition, a regressor (task) encoded whether or not the mouse made a response in that trial, that is, fitting activity related to task engagement. One regressor set aligned to running onset (run), the first imaging frame in a trial in which running speed exceeded 1 cm per second. We implemented two choice-related regressor sets: one aligning with the first sequence of three licks in a row on the side where the mouse would also choose to lick in the response window (choice left/right 1), the other aligning with the first lick during the response window (choice left/right 2), which was the decisive lick in the behavioral paradigm. One regressor set (reward) aligned to reward occurrence, and one (T.O.) to the moment that the time-out was given. Two continuous regressor sets were constructed from the per-imaging-frame lick rate of the mouse, that is, lick rate (left) and lick rate (right), and one continuous regressor set reflected the running speed of the mouse. Finally, we added a constant offset to the model.

All regressor sets were combined in a single design matrix (Fig. 5a). The response variable, inferred spike activity, was smoothed with a Gaussian kernel (*σ* = 0.5 s). The data were subsequently divided in individual trials, only including data that were in range of at least one trial-aligned discrete regressor set. Model parameters were fit on a subset of trials (70%) using NNLS fitting (SciPy) and L1 regularization (where L1 = 0.1 × size of response variable). This specific L1 value was determined by comparing trained model fitted *R*^2^ values with cross-validated *R*^2^ values using a wide range of possible L1 values (Extended Data Fig. 7a). Model performance was expressed as *R*^2^ (equations 1–3, where *N* equals the number of imaging frames in the to-be-predicted response variable *y*, and *p*_*i*_ is the frame-by-frame model prediction). Cross-validated model performance (*R*^2^) was calculated on the remaining 30% of trials.

1 $${{\mathrm{SS}}_{\rm{residual}}} = \mathop {\sum }\limits_{i = 1}^N \left( {p_i - y_i} \right)^2$$

2 $${{\mathrm{SS}}_{\rm{total}}} = \mathop {\sum }\limits_{i = 1}^N \left( {y_i - \bar y} \right)^2$$

3 $$R^2 = 1 - \frac{{\rm{SS}}_{{\rm{residual}}}}{{\rm{SS}}_{{\rm{total}}}}$$

Regressor sets were assigned into seven subgroups (Supplementary Table 3) and subgroup unique contribution to the explained variance was calculated by subtracting the *R*^2^ value of the model with regressors belonging to that subgroup shuffled, from the *R*^2^ of the full model, thus resulting in a Δ*R*^2^ value, similar to what is described in ref. ^113^. The Δ*R*^2^ would assume a value of 0 if all the variance that the subgroup explains can also be explained by any combination of regressors from other subgroups. A positive value for Δ*R*^2^ reflects the degree of explained variance that can only be explained by this specific subgroup. For the subgroups that were most central to our analysis (that is, stimulus, orientation/spatial frequency, category, choice and reward), we calculated the maximum variance inflation factor^114^ across all kernels of each model regressor, for every in-task chronic recording. We found that the value of the variance inflation factor never exceeded 5, and typically ranged between 1 and 4.

*R*^2^ values were compared to values of the same model fitted on trial-shuffled data to establish whether they were significantly above chance, using the following procedure. Chance-level model performance was determined by fitting the model to a trial identity response variable that was shuffled (thus keeping confounders like the nonspecific within-trial temporal structure and offsets the same in the shuffled model). Both the non-shuffled and shuffled model fits were repeated 100 times. We used a non-parametric bootstrap procedure^115^ to estimate the mean and 95% confidence interval of the cross-validated *R*^2^ values of both the shuffled and non-shuffled models. An *R*^2^ value was considered significant if (1) the mean of the shuffled *R*^2^ value was below the lower 95% confidence interval of the non-shuffled R^2^ value, and (2) the mean of the non-shuffled *R*^2^ value was above the upper 95% confidence interval of the shuffled *R*^2^ value.

A semantic CTI was calculated from weights of the left-category and right-category regressor sets (equation (4); where $\bar w_L$ is the mean of all left-category regressor weights across the cross-validation trials, and $\bar w_R$ is the same for right-category weights). Feature CTI was calculated from the weights of the orientation and spatial frequency-specific regressors selectively (equation (5)).

4 $${{\rm{Semantic}}\;{\rm{CTI}}} = \frac{{\bar w_L - \bar w_R}}{{\bar w_L + \bar w_R}}$$

5 $${{\rm{Feature}}\;{\rm{CTI}}} = \frac{{\mathop {\sum }\nolimits_l^L \left( {\bar w_{ori_l} + \bar w_{sf_l}} \right) - \mathop {\sum }\nolimits_r^R \left( {\bar w_{ori_r} + \bar w_{sf_r}} \right)}}{{\mathop {\sum }\nolimits_l^L \left( {\bar w_{ori_l} + \bar w_{sf_l}} \right) + \mathop {\sum }\nolimits_r^R \left( {\bar w_{ori_r} + \bar w_{sf_r}} \right)}}$$

Here, *L* is the number of left-category stimuli and *R* is the number of right-category stimuli. $\bar w_{ori_l}$ is the mean of all orientation regressor weights across cross-validations for left-category stimulus *l* and $\bar w_{sf_l}$ is the mean of all spatial frequency regressor weights across cross-validations for left-category stimulus *l*. $\bar w_{ori_r}$ and $\bar w_{sf_r}$ are the same, but for the right category (Fig. 6a).

### Statistics

Statistical analyses were performed using Python (3.7.10), Numpy (1.16.4) and Scipy (1.5.2). No statistical methods were used to predetermine sample sizes, but our sample sizes are similar to those reported in previous publications^47,69,113^. No data were excluded from the experiment involving touch screen operant chambers. We excluded five animals from the experiment involving head-fixed conditioning because they did not reach criterion on the stimulus discrimination task, three animals because their performance dropped to chance level during category learning, and one animal because it refused to lick on the left lick spout. We excluded three animals from the chronic imaging experiment because their cranial windows did not allow imaging at the time point of category learning. Data collection and analysis were not performed blind to the conditions of the experiments. All data are presented as mean (±s.e.m.) unless otherwise noted. Frequency observations were compared using a chi-squared test. Tests for normality of distributions were not conducted, as the number of observations was often below ten, and testing for normality would be underpowered. Thus, behavioral and imaging data were compared using non-parametric tests: a WMPSR test for paired samples, a Mann–Whitney *U* test for independent samples, and a Kruskal–Wallis test, followed by post hoc WMPSR tests or Mann–Whitney *U* tests, when more than two groups were compared. Significance of *R*^2^ values of individual neurons was determined using non-parametric bootstrap procedures as described above.

### Reporting Summary

Further information on research design is available in the Nature Research Reporting Summary linked to this article.

## Online content

Any methods, additional references, Nature Research reporting summaries, source data, extended data, supplementary information, acknowledgements, peer review information; details of author contributions and competing interests; and statements of data and code availability are available at 10.1038/s41593-021-00914-5.

## Supplementary information

Supplementary Information Supplementary Tables 1–3

Reporting Summary

## Data Availability

Data supporting this study are available on https://gin.g-node.org/pgoltstein/category-learning-visual-areas/.

## Code availability

The Python code used for data analysis and production of figures is available on https://github.com/pgoltstein/category-learning-visual-areas/. Custom-written MATLAB and Python routines used for data collection and data preprocessing are available upon reasonable request.
