# Supplementary material for: Mouse visual cortex areas represent perceptual and semantic features of learned visual categories
Source: Nat Neurosci. 2021 Sep 20;24(10):1441–51. doi: 10.1038/s41593-021-00914-5 (PMC8481127; doi:10.1038/s41593-021-00914-5)
Supplement: Supplementary file 1 — Supplementary Tables 1–3 [file 41593_2021_914_MOESM1_ESM.pdf]

nature portfolio

https://doi.org/10.1038/s41593-021-00914-5

Supplementary information

Mouse visual cortex areas represent perceptual and semantic features of learned visual categories

In the format provided by the authors and unedited

Supplementary information for

Mouse visual cortex areas represent perceptual and semantic features of learned visual categories

Pieter M. Goltstein, Sandra Reinert, Tobias Bonhoeffer, & Mark Hübener

Includes:

Supplementary Tables 1–3

Supplementary Table 1 | Number of chronically recorded neurons per mouse and visual cortical area across all in-task and out-of-task imaging time points.

	V1	LM	AL	RL	AM	PM	LI	P	POR
M13	349	296	294		402				
M14	368			273					
M15	358		284					359	267
M16	287	315	322			437			330
M17	435	297	383			432			233
M18	301			260					280
M19	417			364			261	323	
M20		305		299		399			401
M21				341	384	520			182
M22				187		396	345		333
# neurons	2515	1213	1283	1724	786	2184	606	682	2026
# mice	7	4	4	6	2	5	2	2	7

Total # of chronically recorded neurons: 13019

Supplementary Table 2 | Number of chronically recorded neurons per mouse and visual cortical area across in-task imaging time points only.

	V1	LM	AL	RL	AM	PM	LI	P	POR
M13	399	312	309		410				
M14	383			298					
M15	376		308			324		378	298
M16	315	325	323			450			335
M17	441	308	387			447			237
M18	310			273					286
M19	443			382			282	359	
M20		342		332		412			428
M21				348	398	543			265
M22				214		405	352		342
# neurons	2667	1287	1327	1847	808	2581	634	737	2191
# mice	7	4	4	6	2	6	2	2	7

Total # of chronically recorded neurons: 14079

Supplementary Table 3 | Regressors in the encoding model

Name	Aligned to trial event	Range (s)	Group
Discrete regressors			
Orientation 1	Stimulus onset (SO)	-0.5 to +2.5	Stim. all; Ori/SF
Orientation 2	Stimulus onset	-0.5 to +2.5	Stim. all; Ori/SF
Orientation 3	Stimulus onset	-0.5 to +2.5	Stim. all; Ori/SF
Orientation 4	Stimulus onset	-0.5 to +2.5	Stim. all; Ori/SF
Orientation 5	Stimulus onset	-0.5 to +2.5	Stim. all; Ori/SF
Orientation 6	Stimulus onset	-0.5 to +2.5	Stim. all; Ori/SF
Spatial freq. 1	Stimulus onset	-0.5 to +2.5	Stim. all; Ori/SF
Spatial freq. 2	Stimulus onset	-0.5 to +2.5	Stim. all; Ori/SF
Spatial freq. 3	Stimulus onset	-0.5 to +2.5	Stim. all; Ori/SF
Spatial freq. 4	Stimulus onset	-0.5 to +2.5	Stim. all; Ori/SF
Spatial freq. 5	Stimulus onset	-0.5 to +2.5	Stim. all; Ori/SF
Left category	Stimulus onset	-0.5 to +2.5	Stim. all; Category
Right category	Stimulus onset	-0.5 to +2.5	Stim. all; Category
Task	Stimulus onset	-0.5 to +2.5	Task
Run	Running onset	SO-0.5 to +2.5	Running
Choice left 1	First sequence of three left licks in a row	SO-0.5 to +2.5	Choice
Choice right 1	First sequence of three right licks in a row	SO-0.5 to +2.5	Choice
Choice left 2	First left lick in the trial response window	SO-0.5 to +2.5	Choice
Choice right 2	First right lick in the trial response window	SO-0.5 to +2.5	Choice
Reward	Reward delivery	-0.5 to +2.5	Reward
T.O.	First lick in the trial response window in incorrect trials	-0.5 to +2.5	Reward
Continuous regressors			
Lick rate (left)	-	-1.0 to +1.0	Choice
Lick rate (right)	-	-1.0 to +1.0	Choice
Speed	-	-1.0 to +1.0	Running

Overview of regressors included in the encoding model (GLM analysis). The range of the regressor sets is defined relative to the trial event that the regressor was aligned to, except for specific values that are displayed as SO-##. The SO-## ranges started all ~0.5 seconds before stimulus onset. However, also these regressors were always aligned to their associated event (which was defined to be at precisely time point 0 within the range). Note that Left and Right category were defined as the trained categories.
