# Supplementary material for: Mouse visual cortex areas represent perceptual and semantic features of learned visual categories
Source: Nat Neurosci. 2021 Sep 20;24(10):1441–51. doi: 10.1038/s41593-021-00914-5 (PMC8481127; doi:10.1038/s41593-021-00914-5)
Supplement: Supplementary file 2 — Reporting Summary [file 41593_2021_914_MOESM2_ESM.pdf]

nature research

Corresponding author(s): Pieter Goltstein
Mark Hübener

Last updated by author(s): Jun 15, 2021

Reporting Summary

Nature Research wishes to improve the reproducibility of the work that we publish. This form provides structure for consistency and transparency in reporting. For further information on Nature Research policies, see our Editorial Policies and the Editorial Policy Checklist.

Statistics

For all statistical analyses, confirm that the following items are present in the figure legend, table legend, main text, or Methods section.

n/a	Confirmed	
☐	☒	The exact sample size (*n*) for each experimental group/condition, given as a discrete number and unit of measurement
☐	☒	A statement on whether measurements were taken from distinct samples or whether the same sample was measured repeatedly
☐	☒	The statistical test(s) used AND whether they are one- or two-sided<br>*Only common tests should be described solely by name; describe more complex techniques in the Methods section.*
☐	☒	A description of all covariates tested
☐	☒	A description of any assumptions or corrections, such as tests of normality and adjustment for multiple comparisons
☐	☒	A full description of the statistical parameters including central tendency (e.g. means) or other basic estimates (e.g. regression coefficient) AND variation (e.g. standard deviation) or associated estimates of uncertainty (e.g. confidence intervals)
☐	☒	For null hypothesis testing, the test statistic (e.g. *F*, *t*, *r*) with confidence intervals, effect sizes, degrees of freedom and *P* value noted<br>*Give P values as exact values whenever suitable.*
☒	☐	For Bayesian analysis, information on the choice of priors and Markov chain Monte Carlo settings
☒	☐	For hierarchical and complex designs, identification of the appropriate level for tests and full reporting of outcomes
☐	☒	Estimates of effect sizes (e.g. Cohen's *d*, Pearson's *r*), indicating how they were calculated

Our web collection on statistics for biologists contains articles on many of the points above.

Software and code

Policy information about availability of computer code

Data collection	Data were collected using Matlab versions 2014 to 2017 and LabView versions 2012 and 2016. The software 'Scanimage version 4' (Vidrio Technologies) was used to acquire two-photon microscopy data. Visual stimuli were presented using Psychophysics Toolbox extensions (version 3). Other software was custom written and will be shared upon request, noting that this is not production environment ready code and will require knowledge of the Matlab and LabView platforms.
Data analysis	Data analysis was done using Matlab versions 2015 to 2017 and Python version 3.7.10 (using Numpy version 1.16.4 and Scipy version 1.5.2). Analysis code from external sources for image registration (dftregistration.m version 2007), spike inference (constrained_foopsi.m version 2015), artificial neural networks (Tensorflow version 0.7) and video tracking (Deeplabcut version 2.2) is cited in the Methods section. Custom written Matlab and Python routines used for data collection and data preprocessing are available upon reasonable request. The Python code used for data analysis and producing figures is available on https://github.com/pgoltstein/category-learning-visual-areas.

For manuscripts utilizing custom algorithms or software that are central to the research but not yet described in published literature, software must be made available to editors and reviewers. We strongly encourage code deposition in a community repository (e.g. GitHub). See the Nature Research guidelines for submitting code & software for further information.

Data

Policy information about availability of data

All manuscripts must include a data availability statement. This statement should provide the following information, where applicable:
- Accession codes, unique identifiers, or web links for publicly available datasets
- A list of figures that have associated raw data
- A description of any restrictions on data availability

Data supporting this study are available on https://gin.g-node.org/pgoltstein/category-learning-visual-areas).

Field-specific reporting

Please select the one below that is the best fit for your research. If you are not sure, read the appropriate sections before making your selection.

☒ Life sciences ☐ Behavioural & social sciences ☐ Ecological, evolutionary & environmental sciences

For a reference copy of the document with all sections, see nature.com/documents/nr-reporting-summary-flat.pdf

Life sciences study design

All studies must disclose on these points even when the disclosure is negative.

Sample size	No statistical methods were used to pre-determine sample sizes but our sample sizes are similar to those reported in previous publications (see e.g. refs 47,113).
Data exclusions	No data were excluded from the experiment involving touch screen operant chambers. We excluded five animals from the experiment involving head-fixed conditioning because they did not reach criterion on the stimulus discrimination task, three animals because their performance dropped to chance level during category learning, and one animal because it refused to lick on the left lick spout. We excluded three animals from the chronic imaging experiment because their cranial windows did not allow imaging at the time point of category learning.
Replication	We have replicated the basic behavioral category learning experiment, which we initially carried out using a touch screen operant chamber paradigm, by subsequently employing a head-fixed two-alternative choice task. Basic head-fixed category learning experiments were acquired in two batches, in each batch approximately 50% to 70% of all animals reached the final task and performed well. Experiments probing the retinotopic selectivity of the learned association were performed in two batches, each showing the same effect. For the imaging data, we have approached the finding of areal specialization using two different analysis approaches. One focusing on the average response of neurons during stimulus presentation, and one approach using a linear model to fit and predict the neuronal responses across the entire trial in detail. The results of these analyses corresponded well to each other. However, the entirety of the chronic imaging experiment, as well as the cortical inactivation experiment, has only been performed once and was thus not replicated: The main reason being that this would have taken another two-three years to complete.
Randomization	The information integration stimulus spaces and category boundaries were assigned randomly to individual animals, under the condition that all permutations of stimulus spaces and category boundaries were used roughly equally across animals.
Blinding	Experimenters were not blinded to experimental conditions, as the experimental conditions required specific actions from the experimenter (such as selecting the stimulus space, positioning the computer monitor, and/or imaging cells in a specific visual area). Analyses were not explicitly performed in a blinded fashion, but the scale of the dataset resulted in the experimenter being unaware of group assignment of individual data points during non-automatized procedures (such as ROI selection and matching).

Reporting for specific materials, systems and methods

We require information from authors about some types of materials, experimental systems and methods used in many studies. Here, indicate whether each material, system or method listed is relevant to your study. If you are not sure if a list item applies to your research, read the appropriate section before selecting a response.

Materials & experimental systems

n/a	Involved in the study	
☒	☐	Antibodies
☒	☐	Eukaryotic cell lines
☒	☐	Palaeontology and archaeology
☐	☒	Animals and other organisms
☒	☐	Human research participants
☒	☐	Clinical data
☒	☐	Dual use research of concern

Methods

n/a	Involved in the study	
☒	☐	ChIP-seq
☒	☐	Flow cytometry
☒	☐	MRI-based neuroimaging

Animals and other organisms

Policy information about studies involving animals; ARRIVE guidelines recommended for reporting animal research

Laboratory animals	We used adult male C57Bl/6 mice ranging from 6 to 10 weeks of age at the start of the experiment, except for in a subset of experiments (stimulus-shift experiment, n=3; local cortical inactivation experiment, n=3). There we used mice that express GCaMP6s in excitatory neurons (B6;DBA-Tg(tetO-GCaMP6s)2Niell/J, Jax #024742, crossed with B6.Cg-Tg(Camk2a-tTA)1Mmay/DboJ, Jax #007004; Mayford et al., 1996; Wekselblatt et al., 2016). These mice (two female, one male) were between 12 and 15 weeks of age at the onset of the experiment.

Wild animals	No wild animals were used in this study.
Field-collected samples	No field-collected samples were used in this study.
Ethics oversight	All experimental procedures were done according to institutional guidelines of the Max Planck Society and the regulations of the local government ethical committee (Beratende Ethikkommission nach §15 Tierschutzgesetz, Regierung von Oberbayern).

Note that full information on the approval of the study protocol must also be provided in the manuscript.
